# An integrated framework TSV-INet for arbitrarily distributed TSV interposer wafer warpage simulation

**DOI:** 10.1038/s41378-026-01352-8

**Published:** 2026-07-02

**Authors:** Hanwen Cui, Xiaoyue Ding, Yanze Gao, Xuhao Wan, Tianjian Liu, Yunyun Sun, Huai Zheng, Yikang Zhou, Kai Zheng, Zhiliang Xia, Zongliang Huo, Yuzheng Guo, Sheng Liu, Zhaofu Zhang

**Affiliations:** 1 School of Integrated Circuits, Wuhan University, Wuhan, 430072 China; 2 School of Power and Mechanical Engineering, Wuhan University, Wuhan, 430072 China; 3 NOMAD Laboratory, Fritz Haber Institute of the Max Planck Society, Berlin, 14195 Germany; 4 Yangtze Laboratory, Wuhan, 430205 China; 5 Semiconductor Technology Innovation Center (Beijing) Corporation, Beijing, 100176 China; 6 Yangtze Memory Technologies Co., Ltd, Wuhan, 430205 China

**Keywords:** Electrical and electronic engineering, Other nanotechnology

## Abstract

Through-silicon vias (TSVs) are densely integrated in TSV interposer wafers, and TSV-induced warpage can degrade downstream manufacturing yield. Fast, accurate design-stage warpage simulation is therefore essential. This study presents TSV-INet, a hybrid framework that combines a convolutional neural network and a graph neural network to predict the anisotropic effective properties of TSV representative volume elements (RVEs) and enable wafer-level warpage simulation through RVE-based finite element homogenization. By combining pixel-level material-field encoding with topology-aware message passing on TSV layout graphs, TSV-INet improves data efficiency and robustness to previously unseen layouts relative to CNN-only baselines. Using this framework, we further examine the effects of TSV layout and density on wafer-level thermo-mechanical behavior. For non-extreme layouts with identical TSV count, layout redistribution has only a limited influence on global warpage, but it substantially affects local stress concentration within the interposer die. By contrast, increasing TSV density markedly amplifies wafer warpage and promotes the transition from bowl-like to saddle-like deformation. These findings provide practical guidance for warpage-aware and stress-aware TSV interposer design.

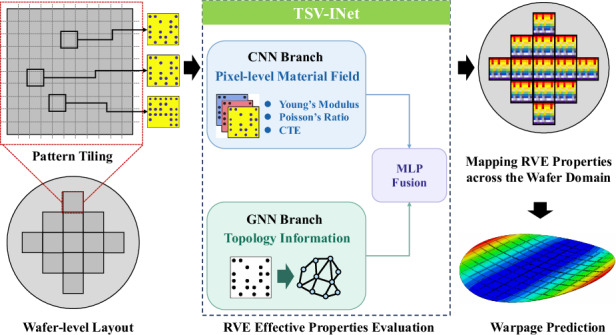

## Introduction

With the continuous shrinkage of interconnect density, the cost and manufacturing difficulty of substrates with finer pitches are escalating. To meet the surging demands of high-performance computing applications, Through-silicon via (TSV) interposers have emerged as a promising solution^1–3^. TSV interposers are carriers fabricated via semiconductor technology, which are typically placed between the dies and package substrates in integrated chips to leverage their advantages in interconnect densities, form factors, and heat dissipation^4,5^. This advanced packaging technology has been applied as 2.5D^6,7^ and 3.5D integrated circuit (IC) packaging^8^.

TSV interposers achieve higher interconnect density by integrating high-density TSV arrays and multi-layer re-distribution layers (RDLs). Owing to the metal grain growth in electroplated Cu within TSV fillings and RDL traces and the pronounced thermal expansion coefficient (CTE) mismatch between heterogeneous materials^9,10^, TSV wafers exhibit various degrees of warpage when the temperature of the process environment changes. Excessive wafer warpage can severely disrupt the continuity of the entire process, while also adversely impacting product yield and long-term reliability^11–13^. Currently, with the continuous miniaturization of TSV and the constant expansion of the silicon interposer integration area^14^, the density of copper per unit volume in TSV wafers increases significantly. This trend has amplified the influence of TSVs on wafer warpage. Conducting preliminary simulation and prediction of the warpage level for a specifically designed TSV wafer is a key link in the warpage optimization. Even among geometries with similar TSV densities, mechanical responses can vary markedly. Therefore, accurate wafer-warpage modeling using the finite element method (FEM) must account for individual TSV characteristics^15^. However, explicitly resolving every TSV causes the mesh number to explode, making detailed simulation impractical.

The FEM simulation based on representative volume element (RVE) models is the mainstream technical approach for simulating the mechanical behaviors of wafer-level or packaging-level warpage^16^. An RVE-based FEM model partitions the full TSV-wafer layout into material-homogenized unit blocks, replicating the mechanical behavior of the actual TSV patterns. The implementation workflow appears in Fig. S1. The workflow starts by discretizing the back-end of line (BEOL) layers into equal-sized tiles, and RVE homogenization of these tiles yields effective properties that are assigned to their respective blocks. This method eliminates the need for detailed modeling of individual TSVs while ensuring computational efficiency. However, because TSVs are largely randomly distributed across the wafer, redundancy among RVEs is low. In the present wafer-warpage context, the “RVE-based” formulation is used in an engineering sense to denote a local block-wise homogenization strategy for spatially heterogeneous TSV layouts, rather than a single statistically converged RVE in the strict classical micromechanics sense. Traditional RVE workflows must compute effective mechanical properties for tens of thousands of distinct blocks at the wafer level, which is also computationally prohibitive.

To mitigate this inefficiency, Jang et al. developed an intelligent pattern clustering system based on a variational autoencoder (VAE) architecture, which encodes the layout features into a low-dimensional latent representation according to the mechanical similarity and grouping using a Gaussian mixture model^17^, thereby significantly reducing the number of unit blocks that need to be calculated by FEM simulation. Computation time is reduced from an estimated 6944 days to just 3 days for each simulation. Although this approach drastically shortens the turnaround time relative to the traditional RVE method, a multi-day runtime still remains too long for rapid design-stage iteration. Numerous studies have employed convolutional neural networks (CNNs) to directly predict the effective properties for each block on the wafer^18–22^. In these studies, layouts are encoded as binary masks or positional matrices, and CNNs are utilized to extract and identify pattern-specific features. Once trained, such surrogates enable highly efficient, near real-time prediction across all RVEs on a chip. However, achieving reliable accuracy typically requires a large and sufficiently diverse set of high-fidelity labeled datasets. Data generation for model development is often computationally expensive and time-consuming. Moreover, because CNNs are best suited to deal with grid-like data, whereas TSV layouts are inherently sparse point patterns, existing CNN-based surrogates often show limited transferability and robustness under domain shifts, with performance degrading when confronted with layout distributions that differ from the training set. Therefore, there is a clear need for a model that enables real-time inference to support rapid design iteration, while also achieving efficient training and maintaining reliable predictive performance on arbitrary, previously unseen TSV layouts.

In this study, we propose the TSV integrated network (TSV-INet), a hybrid CNN-GNN surrogate framework for predicting TSV-RVE effective properties. Different from existing grid-based surrogate models, TSV-INet incorporates topology-aware representation and global statistical information for sparse TSV layouts, enabling improved data efficiency and generalization to unseen patterns. To the best of our knowledge, this is the first GNN-involved surrogate framework for warpage-oriented TSV-RVE homogenization.

## Results

### The integrated network framework TSV-INet

The thermo-mechanical response of a TSV RVE is primarily determined by the CTE mismatch, metal volume fraction, and TSV spatial distribution. An accurate surrogate model should therefore capture not only local material contrast but also topology-dependent interactions among TSVs. CNNs are well-suited for extracting local features from fixed-resolution material-field images, and have consequently been widely adopted in surrogate modeling of TSV and RDL RVEs^18–20,22^. However, because CNNs rely on grid-based local receptive fields, their ability to explicitly encode interactions between spatially separated TSVs remains limited, which may reduce robustness when the TSV topology deviates substantially from the training distribution.

**Fig. 1:**
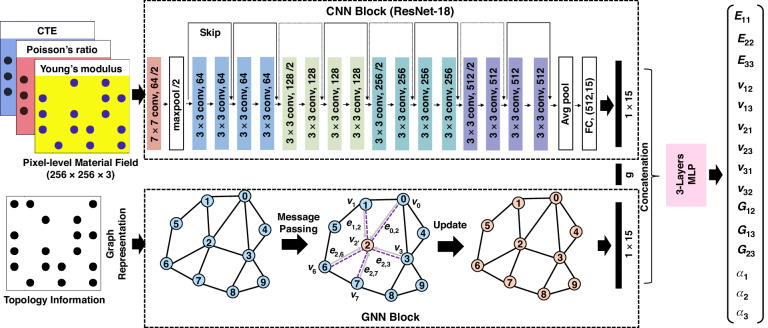
Architecture of the proposed TSV-INet. The CNN branch processes a 256 × 256 three-channel pixel-level material field as input, while GNN branch operates on a symmetrized k-nearest-neighbor graph constructed from the TSV layout. The CNN embedding, GNN embedding, and global descriptor g are fused by a multilayer perceptron to predict the 12 orthotropic elastic constants and the three directional CTE coefficients for each RVE

To address this limitation, TSV-INet introduces a GNN branch that explicitly models the spatial coupling among TSVs. Figure 1 illustrates the overall architecture of TSV-INet. The CNN branch adopts a ResNet-18 backbone to extract a compact material-field embedding from a fixed-resolution three-channel RVE material field, which encodes the pixel-wise difference in Young’s modulus, Poisson’s ratio, and CTE between materials. This encoding directly represents the local property contrast that drives the thermo-mechanical response, providing richer physical information than a binary phase indicator (architectural details in Table S1).

The GNN branch operates on a symmetrized *k*-nearest-neighbor (*k*-NN) graph constructed from the TSV layout. Motivated by the distance-decaying nature of thermo-mechanical interactions, a sensitivity analysis was performed for *k* = 6, 12, and 18, which approximately correspond to progressively expanded local neighborhoods in the staggered TSV arrangement. The performance was found to saturate at *k* = 12, which was therefore adopted in the final model (see Note S3). Each node represents one TSV and carries features describing its geometry, position, and local neighborhood, while edge features encode pairwise spatial relations such as relative distance and orientation (complete feature definitions in Table S2). A GINE-based message-passing network then propagates information across the graph to capture topology-dependent TSV interactions (the update equations are provided in Note S2).

The CNN and GNN branches each produce a feature tensor. These complementary representations are concatenated with the global descriptor *g* and passed through an MLP to predict the 15 orthotropic effective properties (12 elastic constants and 3 directional CTEs). Table S3 provides a systematic comparison of existing approaches and the present work.

#### Comparison with representative mainstream CNN architectures

To assess the advantage of TSV-INet over representative mainstream architectures, we benchmarked it against four widely used CNN-only backbones: VGG-16 (134 M parameters), ResNet-34 (21 M parameters), EfficientNet-B0 (4 M parameters), and MobileNet-V2 (2 M parameters). All models were evaluated on the same fixed out-of-distribution (OOD) test set comprising 53 unique FEM-labeled RVEs with unseen topologies (424 augmented samples), including clustered layouts (with ring/double-ring variants), regular industrial-like layouts, and edge-truncated layouts obtained through spatial translation and clipping (see Note S4). To examine both data efficiency and robustness to topology shift, each model was trained under progressively expanded training-data coverage, and each configuration was repeated in seven independent data splits.

**Fig. 2:**
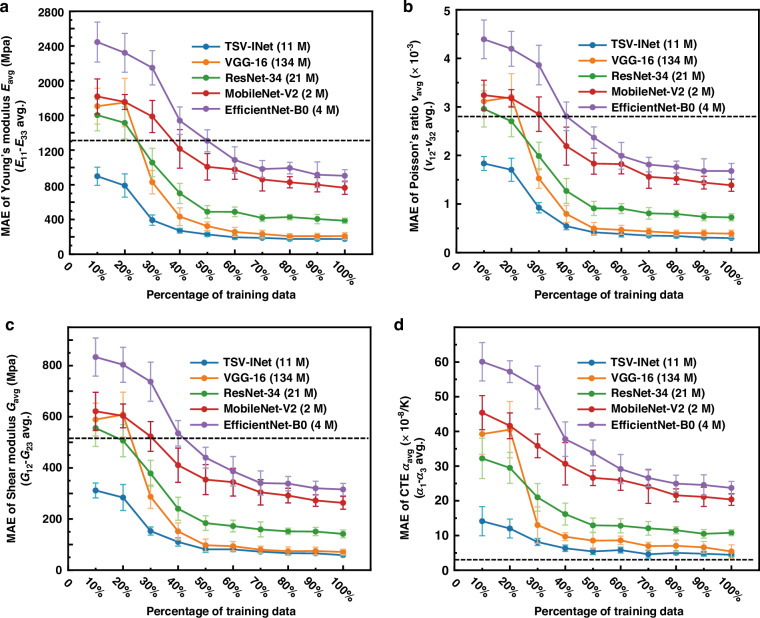
OOD benchmark comparison between TSV-INet and representative CNN-only baselines. **a** Averaged Young’s modulus MAE, **b** averaged Poisson’s ratio MAE, **c** averaged shear modulus MAE, and **d** averaged CTE MAE. Error bars indicate one standard deviation over seven independent data splits. The dashed line marks the 1% relative error referenced to pure-silicon property values. Parameter counts are shown in parentheses

Figure 2 presents the OOD test-set mean absolute error (MAE) for the averaged Young’s modulus, Poisson’s ratio, shear modulus, and CTE as a function of training-data coverage. TSV-INet consistently achieves the lowest MAE across all four quantities and across all data regimes. This advantage is most pronounced in the low-data regime, which is particularly relevant here because the generation of effective-property labels relies on computationally expensive high-fidelity FEM simulations. Notably, for the elastic constants (Fig. 2a–c), TSV-INet falls below the 1% relative-error reference line using only 10–20% of the training data, despite containing only 11 M parameters. In contrast, VGG-16 approaches a comparable mean-error regime only at ~30–40% training-data coverage, and its error bars still overlap the 1% reference line, despite having more than twelve times as many parameters and requiring substantially longer training time. The remaining CNN-only baselines exhibit consistently larger errors. In particular, CTE prediction, the quantity most sensitive to wafer-level warpage (see Note S5), is intrinsically more challenging, and all evaluated models remain above the 1% reference line. Nevertheless, TSV-INet still attains the lowest CTE MAE with the narrowest error bars across nearly all data regimes, indicating both higher accuracy and stronger stability than the CNN-only baselines.

A notable trend is also observed among the CNN-only baselines: with sufficient training data, larger-capacity CNNs tend to outperform lightweight ones in this task, yet all remain inferior to TSV-INet. This suggests that pure CNN surrogates compensate for the lack of explicit topology modeling mainly through increased representational capacity, rather than through a task-matched inductive bias. In natural-image tasks, architectural advances such as depthwise separable convolutions, squeeze-and-excitation attention, and inverted residual blocks are highly effective because the target information is largely texture-driven. By contrast, the thermo-mechanical response of a TSV RVE depends critically on the discrete geometric relations among TSVs as well as global layout statistics. A CNN operating on the pixelized material field can infer these relations only indirectly through hierarchical feature extraction, whereas TSV-INet encodes inter-TSV spatial relations explicitly through graph-based message passing. This difference explains why TSV-INet remains more accurate, more data-efficient, and more robust to topology shift than representative CNN-only architectures.

#### Ablation study of TSV-INet

**Fig. 3:**
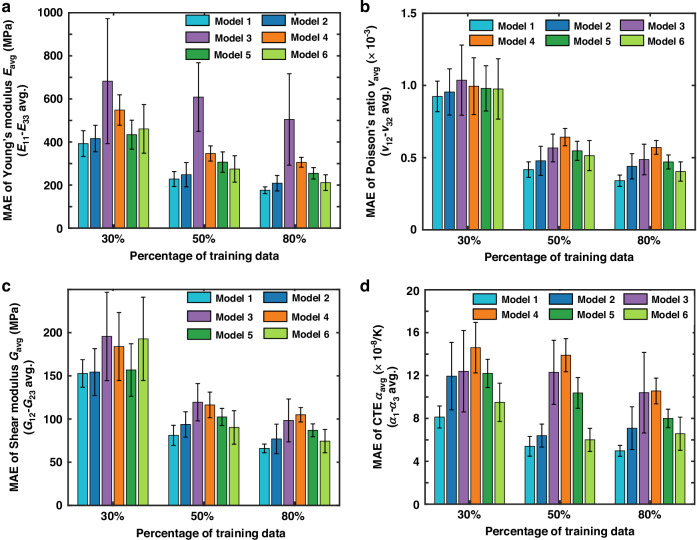
Ablation study results on the OOD test set at 30%, 50%, and 80% training data coverage. **a** Averaged Young’s modulus MAE, **b** averaged Poisson’s ratio MAE, **c** averaged shear modulus MAE, and **d** averaged CTE MAE. Error bars indicate one standard deviation over seven independent data splits. Model configurations are defined in Table 1. Note that Model 1 is the proposed TSV-INet in this work

To further identify the source of the performance gain of TSV-INet, we conducted a systematic ablation study. Six model variants were examined, corresponding to different combinations of the CNN branch, the GNN branch, and the global descriptor *g* (Table 1). Each variant was evaluated under three training-data regimes (30%, 50%, and 80% of the training set) on the same OOD test set. The corresponding results are summarized in Fig. 3. Note that Model 1 is the proposed TSV-INet in this work.

**Table 1:** Component configurations of the six ablation variants

	Model 1	Model 2	Model 3	Model 4	Model 5	Model 6
CNN	√	√	√			√
GNN	√	√		√	√	
Global descriptor *g*	√				√	√

Check marks indicate the inclusion of the CNN branch, GNN branch, and global descriptor *g*

The ablation results indicate that the GNN branch contributes substantially to prediction stability. Models incorporating graph-based topology modeling (Models 1, 2, 4, and 5) generally exhibit smaller variance across repeated runs and training-data regimes than CNN-dominant variants. Physically, this is consistent with the fact that the effective thermo-mechanical response is governed not by individual TSVs in isolation, but by the collective mechanical coupling among neighboring TSVs, including stress shielding, load redistribution, and cooperative thermal-mismatch effects. Message passing on the TSV adjacency graph explicitly encodes these pairwise and higher-order spatial interactions, thereby introducing a topology-aware inductive bias that improves both data efficiency and run-to-run stability. In contrast, a CNN operating on the pixelized material field must infer such interactions implicitly through hierarchical feature extraction, without an explicit structural prior on where the mechanically relevant relations reside, which makes CNN-only models more sensitive to training variation under OOD evaluation.

However, without the pixel-level material-field encoding provided by the CNN branch, the GNN-only model (Model 4) shows a clear accuracy ceiling, indicating that topology-aware modeling alone is insufficient for high-fidelity prediction. The GNN captures the discrete spatial arrangement of inclusions, but does not resolve the continuous material-field gradients that also influence the effective properties. The CNN branch supplies this complementary information by extracting dense, spatially resolved features from the material-field image. Furthermore, integrating the global descriptor *g* consistently improves prediction accuracy, especially for CTE-related quantities. This suggests that graph-level layout statistics provide useful complementary information beyond local material-field and pairwise topology features, serving as a compact summary of the macro-scale configurational context that modulates the overall mechanical behavior of the RVE.

The best performance is achieved only when the CNN branch, GNN branch, and global descriptor are used jointly. This demonstrates that the superiority of TSV-INet arises from the synergistic integration of local material-field encoding, topology-aware graph modeling, and global layout descriptors, rather than from any single component alone. This comprehensive improvement is important for downstream wafer-warpage simulation because different effective properties influence warpage magnitude and deformation mode with different sensitivities, as discussed in Note S5.

### Validation of TSV-INet for RVE-based homogenization

In this section, TSV-INet for RVE-based homogenization is evaluated by comparing its performance against full-model FEM simulations that resolve every individual TSV structure. Three different TSV test dies with different footprints and arbitrary layouts are considered: 1.92 mm 1.92 mm (5496 TSVs), 2.40 mm 2.40 mm (8692 TSVs), and 2.88 mm 2.88 mm (12528 TSVs), and the three test dies are named Case A, B, and C, respectively. All TSVs had a uniform diameter of 10 μm, the die thickness was 30 μm, and the material properties are listed in Table S4. The TSV density distributions of the three validation cases are shown in Fig. S2. To improve computational efficiency, all three test dies were simulated using the 1/4 models based on their geometric symmetry.

**Fig. 4:**
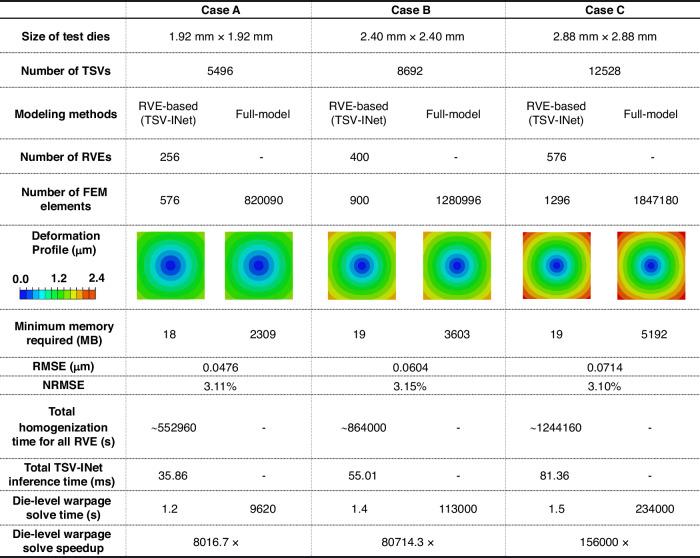
Validation of TSV-INet-based RVE homogenization against full-model FEM for three TSV test dies with arbitrary layouts (Fig. S2). The figure compares model complexity, computational time, and deformation prediction accuracy. The deformation row shows the room-temperature deformation profiles for Cases A–C. 2D top-view error elevation maps are provided in Fig. S4

For the RVE-based homogenization, each layout of the test dies was discretized into RVE blocks using 16$\times$16, 20$\times$20, and 24$\times$24 grids, yielding 256, 400, and 576 RVEs, respectively. Figure S3 compares the anisotropic CTE maps calculated by FEM with those predicted by TSV-INet across TSV dies. Figure 4 compares the mesh count, computational time, and deformation prediction error between the two modeling methods. The proposed framework closely reproduces the full-model predictions across all three cases, achieving NRMSE values of 3.11%, 3.15%, and 3.10%. The error distribution across the entire die surface is shown in Fig. S4. The computational acceleration arises from two distinct mechanisms.

The primary mechanism is the mesh reduction enabled by RVE-based homogenization. By replacing the fine, unstructured meshes required to resolve individual TSV geometries with regular, coarse elements carrying homogenized orthotropic properties, the element count is reduced by approximately three orders of magnitude, allowing the die-level FEM solve to complete in 1–2 seconds. This reduction benefits computation in two ways: the drastically smaller global stiffness matrix accelerates both assembly and solving, and the regular element geometry improves numerical integration efficiency. For wafer-level simulations involving multiple dies, full-model FEM becomes computationally prohibitive, whereas the RVE-based approach remains tractable.

The second mechanism is the replacement of per-RVE FEM homogenization with TSV-INet inference. Conventional FEM homogenization requires ~36 minutes per RVE, making the total homogenization cost for a single die comparable to or exceeding the full-model FEM solve time, particularly for dies with spatially heterogeneous layouts where each RVE contains a unique TSV configuration. This severely limits the applicability of conventional RVE methods in advanced packaging warpage simulation. TSV-INet reduces this step to approximately 0.14 ms per RVE, completing all evaluations in 36 ms, 55 ms, and 81 ms for Cases A to C. Therefore, the two mechanisms make the overall workflow practically viable for simulating TSV interposer warpage.

Because the validation above is performed on a fixed RVE discretization for each test die, an additional sensitivity study was carried out to verify that the conclusions are not an artifact of a partition choice. The resulting warpage and full-field error metrics remain consistent across multiple grid choices, indicating that the proposed framework is robust to reasonable changes in RVE discretization. Detailed results are provided in Note S6.

### Design effects of TSV wafer

The proposed model enables design-driven, wafer-level warpage analysis that is largely impractical with conventional methods. Here, we combine TSV-INet with RVE homogenization to investigate the influence of TSV density and layout on wafer warpage during annealing. Multiple 12-inch silicon TSV-interposer wafers, each containing 112 dies with distinct TSV layouts, are modeled. Each die measures 21.6 mm 21.6 mm and is discretized into 2025 elements (45$\times$45), with one homogenized RVE block assigned to each element. A thermal load from 350 °C to 25 °C is simulated in accordance with the actual annealing process condition^23^. Leveraging the wafer’s symmetry, all simulations were conducted on 1/4 wafer models discretized into only 270,036 elements. This enables complete wafer-level warpage simulations to run within several minutes on a standard desktop computer.

#### Effect of TSV layout on TSV wafer warpage

The impact of TSV layout on wafer warpage is of broad interest in semiconductor packaging. Here, three interposer-die designs with identical TSV count (11,104 per die) but different spatial organizations were considered: a center-concentrated pattern (Case X), a perimeter-clustered pattern (Case Y), and a uniform pattern across the die (Case Z), as shown in Fig. S5. These three cases were selected as representative layout archetypes spanning center-biased, edge-biased, and weakly biased in-die TSV organizations.

**Fig. 5:**
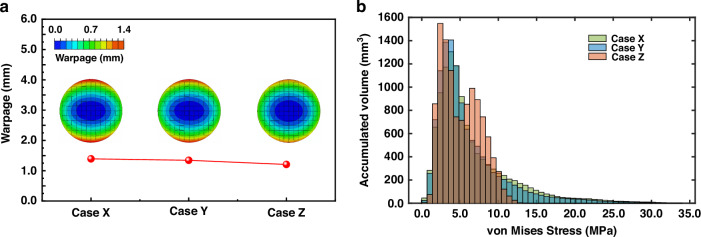
Effect of TSV layout on wafer-level warpage. **a** Maximum warpage for three layout patterns (center-concentrated, perimeter-clustered, and uniform) with identical TSV count (11,104 per die). Inset: representative warpage contours. **b** Cumulative stress-volume distributions for the three layouts, illustrating the divergence in the high-stress regime

Figure 5a compares the wafer-level warpage predicted by TSV-INet for these three layouts. Despite the distinct TSV layouts, the global warpage differs by <15% among the three cases. To decouple wafer-scale bending from die-scale local perturbations, the out-of-plane displacement field was further decomposed using Zernike Polynomials, where low-order terms (*n* $\le$2) represent global bowing and higher-order terms (*n* $>$2) describe localized perturbations^24–26^. The reconstructed profiles agree well with the original displacement fields (Fig. S6). Across all three layouts, the low-order root mean square (RMS) values remain close (0.311, 0.302, and 0.281), and are ~15–16 times larger than the corresponding high-order RMS values (0.019, 0.019, and 0.018). This indicates that wafer warpage is dominated by low-order global bending and is only weakly affected by TSV redistribution within a single interposer die.

In contrast, the local mechanical response shows a much stronger layout dependence. The total strain energy reaches 3.82 mJ for Case X, 3.13 mJ for Case Y, and 1.87 mJ for Case Z. Figure 5b further shows that, although the three layouts exhibit similar stress-volume distributions in the low-stress state, the clustered layouts retain a more pronounced high-stress tail than the uniform layout. This trend is also reflected in the corresponding maximum stresses, which are 39.74 MPa, 40.12 MPa, and 17.02 MPa for Cases X, Y, and Z, respectively. These results indicate that TSV clustering enhances local stress accumulation, whereas uniform spacing mitigates stress concentration (Fig. S7).

Overall, TSV redistribution has only a limited influence on wafer-scale global warpage, but it markedly affects the local stress state and the associated strain energy. This result highlights that TSV-INet is able to distinguish weak layout dependence in global bending from strong layout dependence in local mechanical response.

#### Effect of TSV density on TSV wafer warpage

**Fig. 6:**
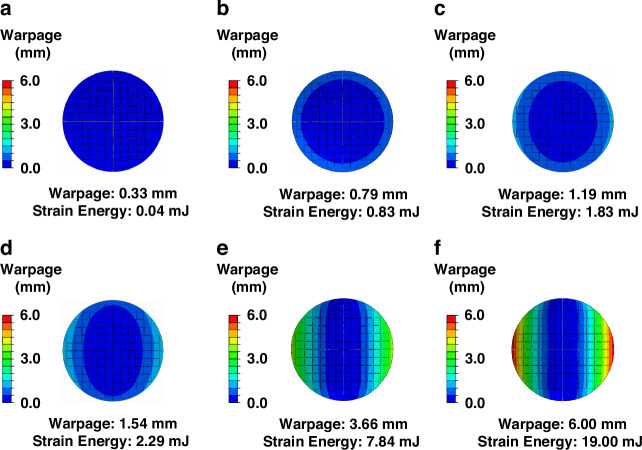
Evolution of warpage deformation across the 12-inch wafer with increasing TSV density on interposer dies with uniform TSV distribution. **a** 2025 TSVs, **b** 7075 TSVs, **c** 11000 TSVs, **d** 12150 TSVs, **e** 20250 TSVs, **f** 30375 TSVs. Maximum warpage and total strain energy are annotated for each case

Increasing the number of TSVs within a single interposer die is a direct route to achieving higher interconnect density. Integrating more TSVs into an interposer die introduces more copper per unit area, which fundamentally alters the wafer’s warpage behavior. In this paper, six wafer-level models were constructed, each integrating interposer dies with a uniform TSV distribution corresponding to Case Z. The TSV count per die was set to 2025, 7075, 11000, 12150, 20250, and 30375, respectively. The resulting wafer warpage after annealing is shown in Fig. 6, and the corresponding von Mises stress field is provided in Fig. S8. Both warpage and strain energy increase systematically with TSV density, indicating that denser TSV integration exacerbates the effective thermo-mechanical mismatch within the wafer. Specifically, the maximum warpage rises from 0.33 mm at 2025 TSVs to 6.00 mm at 30375 TSVs, while the total strain energy increases from 0.04 mJ to 19.00 mJ. This monotonic trend indicates that the increased Cu content per die enhances the thermal mismatch-driven deformation under the same thermal loading.

Beyond the increase in warpage magnitude, the deformation mode also changes qualitatively with TSV density. At low TSV density, the wafer exhibits a predominantly bowl-like warpage, consistent with the previous observations for TSV-integrated wafers^19^. As the TSV density increases, however, the deformation progressively evolves toward a saddle-like pattern, indicating a transition from an axisymmetric bowl-like mode to non-axisymmetric warpage mode. To quantify this transition, a mode-discrimination criterion, Γ, is introduced based on the relative contribution of the astigmatism-dominated and defocus-dominated second-order Zernike coefficients:

1 $$\Gamma =\frac{\sqrt{{a}_{\mathrm{2,2}}^{2}+{a}_{2,-2}^{2}}}{|{a}_{\mathrm{2,0}}|}$$

here, Γ represents the coefficient of the defocus-dominated deformation, while a_2,2_ and a_2,-2_ correspond to the two astigmatism-dominated components. Since these coefficients share the same physical unit, it is a dimensionless parameter by construction. The warpage modes associated with these three coefficients are illustrated in Fig. S9. A value of Γ < 1 indicates that the second-order deformation is defocus-dominated, whereas Γ > 1 indicates that the astigmatism-dominated contribution has become dominant. Therefore, Γ = 1 serves here as a practical mode-discrimination threshold, rather than implying that all cases with Γ < 1 remain ideally bowl-like. Since the wafer-level deformation field is decomposed on the same normalized circular domain using the same Zernike basis, it is not inherently tied to the absolute wafer radius. Different die sizes influence Γ indirectly by altering the wafer-level warpage field and its modal composition. For the low-density cases, it remains far below unity (Γ = 0.0021 for 2025 TSVs and 0.011 for 7075 TSVs), corresponding to a predominantly bowl-like warpage. As the TSV count increases, it rises rapidly toward unity (Γ = 0.341 for 11,000 TSVs and = 0.8089 for 12,150 TSVs), indicating that the deformation is still defocus-dominated but already perturbed by an increasingly strong astigmatic component, such that the wafer progressively departs from the ideal bowl-like mode. When the TSV count reaches 20250, it exceeds unity (Γ = 1.753), indicating that the astigmatism contribution has become dominant and that the wafer develops a clear saddle-like character. At 30,375 TSVs, Γ further increases to 1.923, corresponding to a fully developed saddle-like mode. These results reveal a progressive evolution from bowl-like to saddle-like warpage with increasing TSV density.

**Fig. 7:**
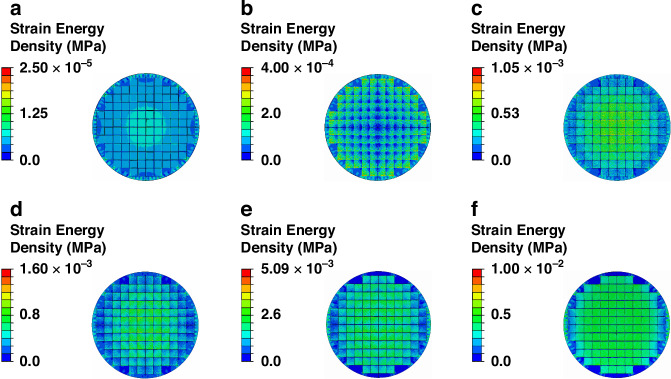
Evolution of strain energy density across the 12-inch wafer with increasing TSV density on interposer dies with uniform TSV distribution. **a** 2025 TSVs, **b** 7075 TSVs, **c** 11000 TSVs, **d** 12150 TSVs, **e** 20250 TSVs, **f** 30375 TSVs. The progressive azimuthal non-uniformity is consistent with the observed bowl-to-saddle mode transition

The strain energy density can characterize the spatial redistribution of elastic energy associated with thermally induced deformation. The strain energy density mapping for these six patterns further elucidates the mechanism underlying the warpage-shape transition, as shown in Fig. 7. With increasing TSV density, the strain energy density not only increases in magnitude but also becomes increasingly azimuthally non-uniform. This change in the energy landscape is consistent with the emergence of non-axisymmetric deformation modes and supports the observed transition from bowl-like to saddle-like wafer warpage.

From a design perspective, these findings indicate that reducing TSV count through higher TSV utilization efficiency is key to mitigating wafer warpage. In contrast, variations in TSV layout have a limited influence on global warpage behavior, provided that the layout remains reasonably uniform and free of extreme patterns.

## Discussion

TSV-INet is a hybrid CNN-GNN framework for efficient TSV-RVE homogenization and wafer-level warpage prediction. Its main advantage lies in the complementary treatment of local material-field information and topology-dependent TSV interactions. The CNN branch resolves pixel-level material contrast, whereas the GNN branch explicitly captures coupling among spatially distributed TSVs through topology-aware message passing. This combination improves robustness to previously unseen layouts while retaining the efficiency required for design-stage evaluation.

The results further distinguish the roles of TSV layout and TSV density in thermo-mechanical behavior. For non-extreme layouts with identical TSV count, layout redistribution has only a limited influence on the global wafer warpage level. However, die-level stress is much more sensitive to layout organization, with clustered TSV patterns producing stronger local stress concentration and higher strain energy than more uniform arrangements. By contrast, TSV density is the dominant factor controlling both wafer-scale deformation and die-level stress, and increasing TSV count also promotes the transition from bowl-like to saddle-like behavior. These observations suggest that layout optimization is mainly important for mitigating localized stress concentration and thereby improving device reliability, whereas total TSV count remains the primary variable for warpage mitigation.

The present framework evaluates deformation driven primarily by thermal-mismatch stress and does not explicitly include process-induced intrinsic stress. In principle, experimentally calibrated residual stress fields, obtained for example via X-ray diffraction, micro-Raman spectroscopy, could be incorporated into the same workflow by applying a pre-stress field in the FEM simulation to improve realism. Likewise, the graph formulation is naturally extensible to more practical scenarios, including defect-aware prediction through additional node or graph-level descriptors such as TSV porosity or seam fraction. An additional implication of this study is that the SiO_2_ insulation layer should not be neglected in homogenization-based TSV warpage analysis, since doing so can overestimate wafer warpage and may shift the apparent transition from bowl-like to saddle-like deformation.

## Material and methods

### TSV-INet training, validation, and testing

TSV-INet and other compared surrogate models were trained on the FEM-labeled RVE dataset described in Note S4, where the details of the coordinate-generation procedure, data augmentation and the construction of the OOD benchmark are provided. The physical treatments of nanoscale functional layers, including the explicitly retained 700 nm SiO_2_ insulation layer, are discussed in Note S5. All descriptors used for model development are summarized in Table S2. Owing to the different magnitudes of the 15 target properties, all labels were min-max normalized before training.

Training was implemented in PyTorch using the AdamW optimizer. Hyperparameters were tuned with Optuna^27^ using the Tree-structured Parzen Estimator (TPE) strategy^28^, and the final model configuration was selected according to the lowest validation loss. The final training setup used a learning rate of 2 × 10^-4^, a weight decay of 2 × 10^−4^, a dropout rate of 0.35, a batch size of 64, and a maximum of 200 epochs. Model optimization was performed using the Huber loss, while the learning rate was scheduled by cosine annealing.

To avoid overfitting and eliminate subjective checkpoint selection, model selection was based solely on the validation loss. Specifically, early stopping was applied with a patience of 20 epochs, a minimum improvement threshold of 10^−4^, and a warm-up period of 10 epochs.

For the comparative benchmark against representative mainstream CNN architectures, each model was evaluated under the same data protocol. In the robustness study, the data split was repeated over seven independent data splits, while the model initialization seed was fixed to isolate the effect of data partitioning from stochastic weight initialization. Performance was then aggregated across these repeated splits.

All FEM simulations and machine learning training were performed on a server equipped with an Intel(R) Xeon(R) Platinum 8336 C CPU (64 cores) and an NVIDIA GeForce RTX 4090 GPU.

### The FEM-based material homogenization

FEM-based material homogenization is discussed in detail in ref. ^29^, and the overall workflow is summarized in Fig. S10 herein. In brief, the effective elastic and CTE tensors are obtained by imposing prescribed macroscopic strain and temperature fields on an RVE and averaging the resulting stress response. The homogenization accuracy critically depends on the boundary conditions^29^, with common choices including Dirichlet, Neumann, and periodic boundary conditions (PBCs). Dirichlet and Neumann conditions are straightforward to implement because they do not require periodic meshing. However, they may induce artificial stress concentrations and thus bias the estimated effective properties^30^. In contrast, PBCs generally yield more reliable homogenized responses^31^. Moreover, for TSV RVEs, effective properties computed under PBCs are reusable across interposers with different TSV aspect ratios.

Traditionally, imposing PBCs requires a one-to-one correspondence between mesh nodes on opposite faces of the RVE to enforce displacement compatibility between matched node pairs. This requirement is difficult to satisfy for RVEs with non-periodic geometries, such as randomly distributed TSV layouts. To accommodate non-matching meshes, a surface-projection strategy is adopted: nodes on the mapping surface are projected onto the opposite target surface (Fig. S11a).

For each projected node S’ on the target surface, a local support domain is defined. The periodic constraint at S’ is imposed by expressing its displacement as a weighted interpolation of the displacements of neighboring nodes within the support domain. The interpolation weights are computed using the radial point interpolation method (RPIM) with radial basis functions (RBFs), a mesh-free technique widely used in both weak and strong-form formulations^32,33^. Owing to its Kronecker-delta property, RPIM can recover the standard node-to-node periodic constraint on periodic meshes while maintaining numerical stability for irregular nodal distributions. For edge nodes, the displacement at the target point N’ is obtained via linear interpolation between its two adjacent nodes N+ and N− (Fig. S11b).

The displacement for the reference node on the surfaces can be expressed by RPIM interpolation as:

2 $$U={R}_{0}A+{P}_{m}B$$

where P_m_ refers to polynomial basis functions of all the reference nodes in 2D coordinates, and R_0_ denotes the RBFs expressed in terms of the Euclidean distance *r* between periodic images and their support nodes. Here, the unknown parameters are solved for, which yields the displacement expression for each reference node.

In order to get and ${\boldsymbol{B}}$, another *m* equations should be added:

3 $$\widetilde{{\boldsymbol{U}}}=\left[\begin{array}{cc}{{\boldsymbol{R}}}_{{\bf{0}}} & {{\boldsymbol{P}}}_{{\boldsymbol{m}}}\\ {{\boldsymbol{P}}}_{{\boldsymbol{m}}}^{{\bf{T}}} & {\bf{0}}\end{array}\right]\left(\begin{array}{l}A\\ B\end{array}\right)$$

The displacement of node S can now be expressed as:

4 $$u({x}_{\mathrm{s}})=\left\{{R}^{\mathrm{T}}({x}_{\mathrm{s}}){P}^{\mathrm{T}}({x}_{\mathrm{s}})\right\}{\left[\begin{array}{cc}{R}_{0} & {P}_{m}\\ {\mathrm{P}}_{m}^{\mathrm{T}} & 0\end{array}\right]}^{-1}\widetilde{U}$$

Multiple RBFs are available; among them, the multi-quadric (MQ) kernel often delivers superior accuracy. The MQ RBF is defined as:

5 $${R}_{i}({\boldsymbol{x}})={[{{\boldsymbol{r}}}_{i}^{2}+{(ad)}^{2}]}^{q}$$

Following Liu et al. a and q were fixed at 8 and 0.98. In addition, the RPIM basis was selected as a quadratic polynomial, and the support domain was chosen as the nine closest nodes, which helps prevent ill-conditioned systems and enhances numerical robustness.

In this work, a Python-based parametric modeling script has been developed to automatically impose PBCs for TSV layouts and to compute the corresponding effective properties. The flowchart of the Python code program is shown in Fig. S12.

### Supplementary information

Supporting Information is available at Appendix A, including Notes S1–S6 (fundamentals of CNNs and GNNs, k-nearest-neighbor ablation and graph-topology statistics, construction of the in-distribution dataset and the OOD benchmark, influence of nanoscale thin films and the SiO_2_ layer, and sensitivity to die-level RVE discretization), Tables S1–S4 (TSV-INet architecture, feature representations, representative related studies and the present work, material properties), and Figs. S1–S12 (workflow of the RVE-based FEM approach, TSV layouts and validation cases, FEM-versus-TSV-INet effective-property comparisons, deformation and error maps, layout- and density-dependent wafer responses, Zernike-based warpage reconstruction, graph-topology statistics, representative OOD layouts, and the FEM homogenization/PBC implementation workflow).

## Supplementary information

Supplementary Information

## Data Availability

The datasets used and analyzed during the current study are available from the corresponding author on reasonable request.
