# Supplementary material for: An integrated framework TSV-INet for arbitrarily distributed TSV interposer wafer warpage simulation
Source: Microsyst Nanoeng. 2026 Jul 2;12:251. doi: 10.1038/s41378-026-01352-8 (PMC13328617; doi:10.1038/s41378-026-01352-8)
Supplement: Supplementary file 1 — Supplementary Information [file 41378_2026_1352_MOESM1_ESM.docx]

Supporting Information

**An Integrated Framework TSV-INet for Arbitrarily Distributed TSV Interposer Wafer Warpage Simulation**

Hanwen Cui*^a^*, Xiaoyue Ding*^a^*, Yanze Gao*^a^*, Xuhao Wan*^b,c^*, Tianjian Liu*^d^*, Yunyun Sun*^b^*, Huai Zheng*^a^*, Yikang Zhou*^e^*, Kai Zheng*^e^*, Zhiliang Xia*^f^*, Zongliang Huo*^f^*, Yuzheng Guo*^b^*, Sheng Liu*^a,b^*, Zhaofu Zhang*^a^*^*^

*a* School of Integrated Circuits, Wuhan University, Wuhan, 430072, China

*b* School of Power and Mechanical Engineering, Wuhan University, Wuhan, Hubei, 430072, China

*c* NOMAD Laboratory, Fritz Haber Institute of the Max Planck Society, Faradayweg 4-6, 14195 Berlin, Germany

*d* Yangtze Laboratory, Wuhan 430205, China

*e* Semiconductor Technology Innovation Center (Beijing) Corporation, Beijing, 100176, China

*f* Yangtze Memory Technologies Co., Ltd, Wuhan 430205, China

* Corresponding Author: [zhaofuzhang@whu.edu.cn](mailto:zhaofuzhang@whu.edu.cn)

**Note S1. The Fundamental Principle of Convolutional Neural Networks.**

The **Convolutional Neural Networks (CNNs)** are a foundational class of deep learning models and remain widely used for learning representations from grid-structured data (e.g., images and fields). In a CNN, convolutional layers extract hierarchical features by applying learnable kernels that are shared across spatial locations, greatly reducing the number of parameters compared with fully connected layers. A typical CNN mining features from input data through convolution layers, which employ a set of kernels to capture spatial patterns from grid-structured inputs. Unlike fully-connected layers, the kernel shares its weights (including bias $\text{b}^{\text{k}}$ and filter weights $\text{w}^{\text{k}}$) across the entire input. Each kernel is designed to produce a specific feature map $\text{m}^{\text{k}}$ for the $\text{k}^{\text{t}\text{h}}$ feature by computing the dot product between its weights and the local input region, as expressed in Eq. S1.

$\text{m}^{\text{k}}\text{=}\text{f}\text{(}\text{w}^{\text{k}}\text{∗}\text{x}\text{+}\text{b}^{\text{k}}\text{)}$ (*S1*)

here, * denotes convolution operation, $\text{w}^{\text{k}}$ and $\text{b}^{\text{k}}$ are the kernel weights and bias, *f*(·) is a nonlinear activation function. Stacking multiple convolution-activation blocks enables the network to capture increasingly complex patterns. Batch normalization is commonly inserted to stabilize feature distributions and accelerate training, while pooling (e.g., max, average pooling) or stride operations down-sample feature maps to reduce spatial resolution and improve robustness. The resulting representations are then aggregated and fed into fully connected layers to produce task-specific outputs for classification or regression.

**Note S2. The Fundamental Principle of Graph Neural Networks.**

CNNs are well-suited for learning from data defined on Euclidean domains, where samples lie on regular grids with a fixed neighborhood structure. Many real-world data, however, are non-Euclidean: the neighborhood size may vary across samples, and there is no canonical ordering of neighbors. Such data are naturally represented as graphs. A graph is defined as ***G =* (*V*, *E*)**, where ***V=*{**$\text{v}_{\text{1}}\text{,}\text{v}_{\text{2}}\text{,⋯,}\text{v}_{\text{n}}$**}** is the node set and ***E*={**$\text{(}\text{v}_{\text{i}}\text{,}\text{v}_{\text{j}}\text{)}$**}** is the edge set. The connectivity can be encoded by an adjacency matrix ***A***$\text{∈}\text{R}^{\text{n}\text{×}\text{n}}$ (binary or weighted), which is typically sparse because each node connects to only a small subset of other nodes. **Graph Neural Networks (GNNs)** are designed to learn directly on graphs by iteratively exchanging information along graph edges while preserving the input connectivity. In each layer, a node forms a message from its neighbors (optionally using edge features), aggregates these messages using a pooling operation (e.g., sum, mean, or max), and then updates its own representation based on the aggregated neighborhood information and its current state. Repeating this process enables GNNs to capture multi-hop dependencies and learn node embedding. For graph-level prediction, node representations can be further pooled into a single vector, enabling end-to-end classification or regression at the node, edge, or graph level.

In TSV-INet, the GNN branch employs the Graph Isomorphism Network with Edge features (GINE) [S1] as the core message-passing operator. GINE extends the standard Graph Isomorphism Network (GIN) [S2] by incorporating edge attributes into the message construction. This is essential for the present application because the pairwise spatial relations between TSVs (e.g., relative distance, orientation) carry physically meaningful information governing the strength of thermo-mechanical coupling. The update rule for node *i* at layer *l* is formulated as:

$\text{m}_{\text{ij}}^{\text{(}\text{l}\text{)}}\text{=ReLU(}\text{h}_{\text{j}}^{\text{l}}\text{+}{\text{Φ}_{\text{e}}^{\text{(}\text{l}\text{)}}\text{e}}_{\text{ij}}\text{) }$(*S2*)

$\text{h}_{\text{i}}^{\text{(}\text{l}\text{+1)}}\text{=}\text{MLP}^{\text{(}\text{l}\text{)}}\text{((1+}\text{ϵ}^{\text{(}\text{l}\text{)}}\text{)}\text{h}_{\text{i}}^{\text{l}}\text{+}\sum_{\text{j}\text{∈}\text{N}\text{(}\text{i}\text{)}} \text{m}_{\text{ij}}^{\text{(}\text{l}\text{)}}\text{)}$ (*S3*)

where, $\text{h}_{\text{i}}^{\text{l}}$ denotes the feature vector of node *i* at layer *l*, $\text{N}\text{(}\text{i}\text{)}$ is the set of neighbor of node *i* (determined by the *k*-nearest-neighbor graph with *k*=12), $\text{Φ}_{\text{e}}^{\text{(}\text{l}\text{)}}$ is the edge encoder, $\text{e}_{\text{ij}}$ is the edge feature vector between node *i* and *j*, $\text{ϵ}^{\text{(}\text{l}\text{)}}$ is a learnable scalar parameter, and $\text{MLP}^{\text{(}\text{l}\text{)}}$ is a multi-layer perceptron applied at each layer. The summation over neighbors serves as the aggregation operator, while the ReLU activation ensures that edge features contribute non-linearly to the message formulation.

After *L* layers of message passing, the node-level representations are aggregated into a unified graph-level embedding via global mean pooling:

$\text{h}_{\text{G}}\text{=}\frac{\sum_{\text{i}\text{∈}\text{V}} \text{h}_{\text{i}}^{\text{(}\text{L}\text{)}}}{\left| \text{V} \right|}$ (*S4*)

This graph-level vector serves as the ultimate output of the GNN branch and is subsequently concatenated with the CNN branch output and the global descriptor *g* for downstream property prediction.

The choice of GINE over alternative message-passing operators (e.g., GCN, GAT) is motivated by two key considerations. First, the standard GIN architecture has been shown to be as powerful as the 1-dimensional Weisfeiler-Leman (1-WL) test in distinguishing a broad class of graph structures. Building upon this idea, GINE retains strong discriminative capability while additionally incorporating edge attributes. Second, the explicit incorporation of edge features in GINE enables the model to natively leverage pairwise geometric information (distance, spatial orientation) without requiring separate edge-level networks.

**Note S3. Ablation Study of *k*-nearest-neighbor in TSV-INet.**

To determine an appropriate neighborhood size for graph construction, we performed an ablation study on the *k*-nearest-neighbor (*k*-NN) graph used in the GNN branch, with *k* = 6, 12, and 18. This choice was guided by the geometric intuition of the parent staggered TSV arrangement used in the coordinate-generation procedure. In an ideal locally hexagonal packing, each TSV has approximately six closest neighbors in the first coordination shell. Therefore, *k* = 6 serves as a physically motivated baseline that captures the most immediate local interactions. Expanding the neighborhood to *k* = 12 allows the graph construction to include not only the nearest shell but also a broader second-layer local context, thereby enabling the GNN to aggregate richer pairwise and higher-order spatial information. A further increase to *k* = 18 was introduced to probe an extended local-to-mid-range regime and to test whether incorporating more distant neighbors can further improve predictive performance. The results are summarized in Table N3.1.

**Table N3.1.** Results of the k-nearest-neighbor ablation study (*k* = 6,12,18)

|  | $\text{MAE}_{\text{E}\text{avg}}$  **(MPa)** | $\text{MAE}_{\text{v}\text{avg}}$  **(×10^-4^)** | $\text{MAE}_{\text{G}\text{avg}}$ **(MPa)** | $\text{MAE}_{\text{CTEavg}}$  **(×10^-8^/K)** |
| --- | --- | --- | --- | --- |
| ***k* = 6** | 179.65$\text{±}$27.54 | 2.99$\text{±}$0.18 | 64.88$\text{±}$7.06 | 4.56$\text{±}$0.37 |
| ***k* = 12** | 174.98$\text{±}$9.27 | 2.97$\text{±}$0.20 | 58.70$\text{±}$3.42 | 4.45$\text{±}$0.30 |
| ***k* = 18** | 182.41$\text{±}$16.3 | 2.99$\text{±}$0.33 | 61.46$\text{±}$5.63 | 4.49$\text{±}$0.39 |

Among the three settings, *k* = 12 achieves the best overall performance, yielding the lowest average MAE for Young’s modulus, Poisson’s ratio, shear modulus, and CTE. In addition, the overall run-to-run fluctuations are generally smaller at *k* = 12 than at *k*=6 and 18, indicating improved training stability. These results suggest that an overly small neighborhood **(***k* = 6**)** may provide insufficient spatial context for the GNN to adequately capture nonlocal thermo-mechanical coupling among TSVs. In contrast, expanding the receptive field to *k* = 12 allows message passing to incorporate richer pairwise and higher-order spatial interactions, thereby providing more effective topology-aware information to complement the local material-field features learned by the CNN branch. This behavior is consistent with the physical intuition that TSV-induced interactions are not limited to the immediately closest neighbors, but can extend to a broader local neighborhood. When the neighborhood size is further increased to *k* = 18, however, the predictive accuracy no longer improves and instead degrades slightly. This trend suggests that overly large neighborhoods may introduce less relevant long-range connections, which dilute the dominant local coupling patterns and reduce the effectiveness of message aggregation. Therefore, *k*=12 provides the best balance between capturing sufficient topological context and avoiding unnecessary information mixing, and was thus adopted in the final TSV-INet model.

To further characterize the topology induced by the adopted symmetrized k-NN graph with *k* = 12, FIG. N3.1 summarizes the edge count distribution and the node-count distribution per graph across the RVE data set. Since each node corresponds to one TSV, the node-count distribution directly reflects the graph-size variation induced by different TSV densities. As shown in FIG. N3.1(b), the graph size remains bounded within approximately 1 to 49 nodes per graph, with the highest concentration in the 25 to 35 range, indicating that the constructed graphs remain well controlled across both sparse and dense layouts. As shown in FIG. N3.1(a), the edge count distribution exhibits a pronounced peak at approximately 20 to 26, with most nodes concentrated in the 20 to 30 range and only a limited right tail extending to about 40. This behavior is physically consistent with the symmetrized *k*-NN construction: because each node first connects to its *k* = 12 nearest neighbors and reciprocal selections are subsequently retained, the effective degree naturally approaches the order of 2*k* when mutual neighbor selection is common, while nodes in locally dense TSV regions may receive additional reciprocal connections from surrounding neighbors. The small number of very low degree nodes is associated only with degenerate ultra-sparse graphs, specifically the rare cases in which the cropped and randomly filtered RVE contains only one TSV. In such cases, the graph contains a single node and therefore no valid neighbor set, leading to degree-zero nodes. This situation is still well defined within the adopted GNN formulation, because the node representation can continue to be propagated through the self-feature term in the GINE update rule even when the neighbor set is empty. Therefore, these isolated nodes do not indicate a failure of the graph construction, but rather reflect the physically admissible existence of extremely sparse RVEs in the data set.


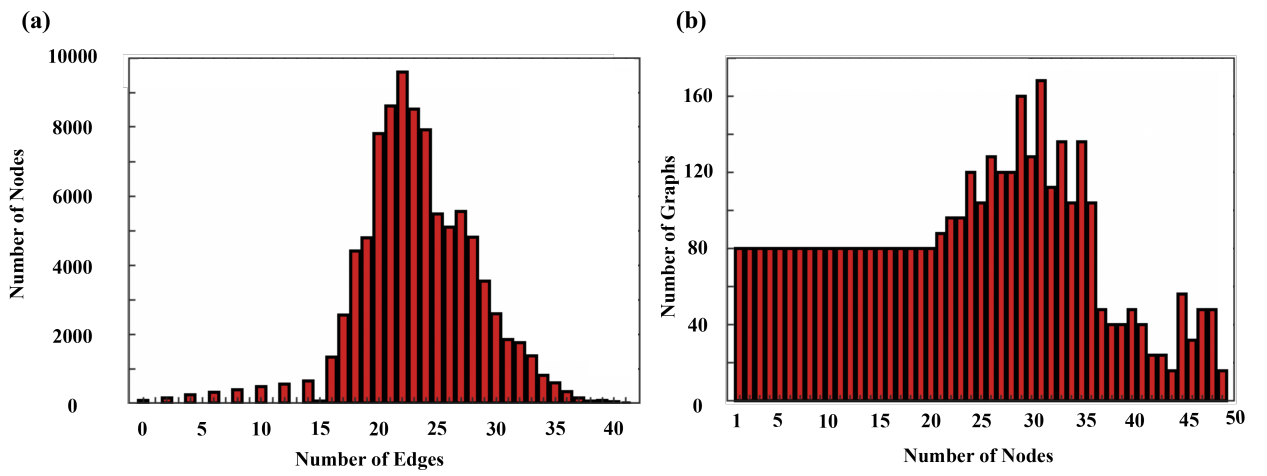


**FIG. N3.1.** Graph topology statistics for the adopted symmetrized *k*-NN graph (*k* = 12). (a) Edge count distribution across all RVE graphs in the data set. The peak at 20 to 26 is consistent with the reciprocal *k*-NN construction (effective degree$\text{ }\text{≈}\text{ }$2*k*). (b) Node-count distribution per graph, reflecting the variation in TSV count across RVEs.

Overall, this ablation supports the role of the GNN branch in explicitly encoding TSV-topology information through message passing on the adjacency graph. The improvement at *k* = 12 indicates that topology-aware aggregation provides a meaningful complement to the CNN pathway, contributing to both higher predictive accuracy and better run-to-run robustness.

**Note S4. Construction of the In-distribution Data Set and the Out-of-Distribution Benchmark.**

1. **Two-stage Random Algorithm to Generate TSV-based RVE**

To construct the in-distribution (ID) data set for model development, TSV layouts were generated using a two-stage random coordinate-generation procedure. The two-stage random process begins with constructing an $\text{8×8}$ staggered TSV array to achieve the densest packing, with the minimum center-to-center pitch of twice the TSV diameter (2*D*_tsv_). This spacing aligns with established rules for ultra-fine pitch TSV wafer and ensures structural integrity by preventing mutual interference during manufacturing and service caused by excessively small spacing.

To balance the computational efficiency while maintaining microstructural diversity, a square RVE domain measuring 12*D*_tsv_ per side is extracted from the center of the staggered array, defining the final TSV RVE boundaries. The randomization procedure subsequently implements two sequential operations:

1. **Array translation and region cropping:** The entire staggered TSV array is randomly translated within the range [0,2*D*_tsv_] in both *x* and *y* directions. After translation, only TSVs that lie entirely within or partially interact with the predefined square RVE boundary are retained.
2. **Random deletion for microstructural diversity:** A random deletion process is applied to the retained TSVs within the square region according to a random target TSV count. This operation further enhances the randomness of TSV distribution, ensuring that the generated RVE samples cover a broader range of microstructural layouts.

This two-stage procedure defines the ID layout distribution used for training, validation, and internal testing.

1. **Construction of the OOD Benchmark.**

The random train/validation/test split described above is appropriate for assessing predictive accuracy within the same generation distribution, but it does not by itself constitute a strong topology-shift test. To evaluate robustness beyond the ID layout family, we therefore constructed a separate out-of-distribution (OOD) benchmark.

In this work, “OOD” refers to TSV layouts whose spatial organization is intentionally shifted away from the two-stage random distribution used for model development. The OOD benchmark includes several explicitly designed topology families (FIG. N4.1), including:

1. **Clustered layouts**, such as center clustered, tight center, four corner clustered, four axis clustered, ring, and double ring patterns.
2. **Regular industrial-like layouts**, such as square array, hexagonal array, and staggered array patterns.
3. **Edge-truncated layouts**, obtained by applying random spatial translation to the above base families followed by clipping with the fixed RVE boundary.

All OOD samples were generated independently of the ID data set and were not used in training, validation, or internal testing.


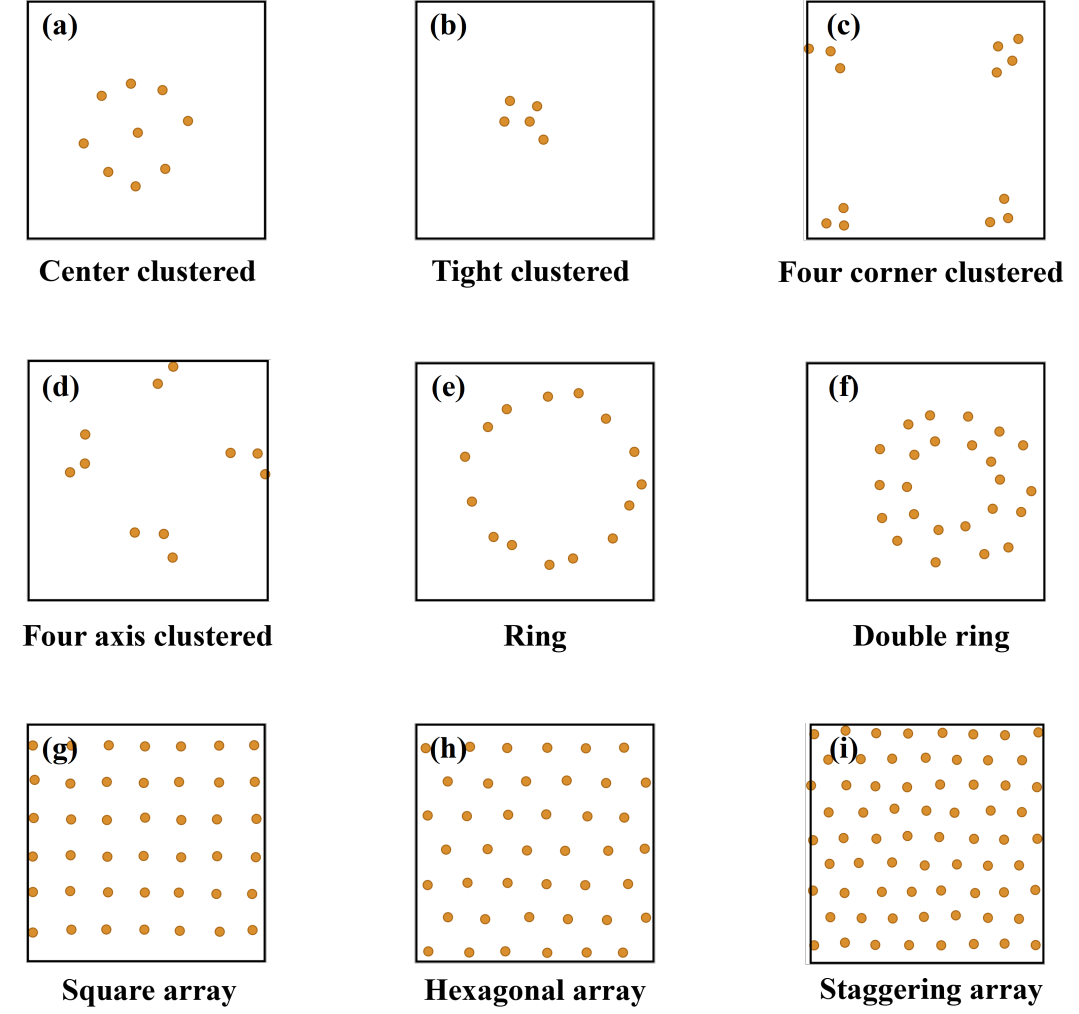


**FIG. N4.1.** Representative OOD benchmark layouts. (a)-(f) Clustered families: center clustered, tight clustered, four corner clustered, four axis clustered, ring, and double-ring patterns. (g)-(i) Regular array families: square array, hexagonal array, and staggered array.

1. **FEM Labeling, Augmentation, and Split Protocol**

Each unique RVE generated by the above procedure was labeled via FEM-based homogenization to obtain 15 effective thermo-mechanical properties (12 orthotropic elastic constants and 3 directional CTEs). Each RVE is a 120$\text{μ}$m$\text{×}$120$\text{μ}$m square containing TSVs of radius 5 $\text{μ}$m with a 700 nm SiO_2_ insulation layer explicitly modeled. This size was chosen as a practical compromise between microstructural representativeness and computational tractability. On the one hand, the 120$\text{μ}$m domain is large enough to accommodate multiple TSVs and their local neighbor interactions within a single block, thereby capturing the mechanically relevant pairwise coupling effects. On the other hand, further increasing the RVE size would substantially increase the FEM element count per RVE and enlarge the graph size in the GNN branch, without necessarily improving the homogenized response, as confirmed by the die-level discretization sensitivity study in Note S6. The labeled data set comprises 500 unique FEM-solved RVEs. To improve data efficiency while preserving physical equivalence, each RVE was augmented by the four rotational (0°, 90°, 180°, 270°) and four reflection symmetries of the square domain[S3], yielding an eight-fold expansion to 4000 augmented training samples. This augmentation is physically exact, the orthotropic effective properties transform deterministically under these symmetry operations, and therefore introduces no approximation error. During training, the 4000 augmented ID samples were split into training and validation sets in a ratio of 8:2.

A separate out-of-distribution (OOD) benchmark was constructed from 53 independently generated unique RVEs featuring topologies absent from the training set: clustered families (16 RVEs, ~30%), ring and double-ring families (16 RVEs, ~30%), and regular array families (21 RVEs, ~40%), with edge-truncated variants included across these base families. After applying the same eight-fold symmetry augmentation, the OOD test set contains 424 samples.

1. **OOD Evaluation Protocol**

All candidate models were evaluated on the same fixed OOD benchmark in order to quantify robustness under topology shift. The primary controlled baseline was a CNN-only ResNet-18, which shares the same backbone as the CNN branch of TSV-INet and therefore isolates the contribution of the graph branch. In addition, representative mainstream CNN architectures, including VGG-16, ResNet-34, EfficientNet-B0, and MobileNet-V2, were also evaluated on the same OOD set. For each training-data regime, all models were trained with the same data protocol and repeated over seven independent data splits. Performance was summarized using the mean absolute error of the predicted effective properties.

$\text{ε}_{\text{MAE}}\text{=}\frac{\sum_{\text{i}\text{=1}}^{\text{n}} \left| \text{y}_{\text{i}}\text{−}\hat{\text{y}_{\text{i}}} \right|}{\text{n}}$ (*S5*)

where, $\text{n}$ is the number of samples, $\text{y}_{\text{i}}$ denotes the FEM-computed target for the *i*^th^ sample, and $\hat{\text{y}_{\text{i}}}$ is corresponding prediction from surrogates.

**Note S5. Assessment of Functional Layers in TSV RVE Homogenization.**

A typical Cu-filled TSV consists, from the center outward, of the Cu filling, a Cu seed layer, a TaN (or Ta/Ti) diffusion barrier, a SiO_2_ insulation liner, and the surrounding Si matrix. Because the present study focuses on homogenized effective properties and wafer-level warpage rather than nanoscale local interfacial stresses, the influence of these thin-film layers should be evaluated in terms of their contribution to the RVE-level constitutive response and the subsequent propagation of that contribution to macroscopic warpage. This note therefore assesses the influence of the nanoscale functional layers from three complementary perspectives: order-of-magnitude estimation based on layer volume fraction, first-order warpage sensitivity analysis, and direct numerical comparison at both the RVE and wafer-levels.

1. **Assessment of the Barrier and Seed Layers**

In standard TSV fabrication, the characteristic thicknesses of the sidewall functional layers are highly disparate: the SiO_2_ insulation liner is typically on the order of 200 to 1000 nm, the Cu seed layer is commonly around 100 nm, and the barrier layer is usually only several tens of nanometers thick[S4,S5]. For a cylindrical TSV of radius *R*, the cross-sectional area fraction of a concentric annular layer with thickness *t* (*t*$\text{≪}$*R*) can be approximated as:

$\text{f}\text{ ≈ 2}\text{t}\text{/}\text{R}$ *(S6)*

Under a Voigt-type iso-strain upper-bound estimate, the contribution of a given layer *i* to an effective composite property scales approximately with its area fraction:

$\text{P}_{\text{eff}}^{\text{Voigt}}\text{=}\sum_{\text{i}} \text{f}_{\text{i}}\text{P}_{\text{i}}$ (*S7*)

so that the first-order contribution of a thin layer is approximately proportional to $\text{f}_{\text{i}}\text{P}_{\text{i}}$. For a 40 nm TaN barrier in a TSV of radius 5 $\text{μ}$m, the corresponding area fraction is only about 1.6%. Even though TaN has a relatively high Young’s modulus, its first-order contribution to the homogenized modulus remains small compared with the overall stiffness scale of the TSV composite. Its corresponding contribution to the effective thermal expansion is likewise limited by the same small geometric fraction. This order-of-magnitude estimate indicates that the barrier layer has only a minor influence on the homogenized properties relevant to wafer-level warpage. This conclusion is also consistent with prior local-stress studies, in which even substantial changes in barrier thickness were reported to induce only very small variations in TSV stress fields. The Cu seed layer differs from the barrier layer in thickness, but not in constitutive role. When the seed layer is assumed to have the same thermo-mechanical properties as the electroplated Cu fill, merging it into the Cu region introduces no meaningful material contrast in the homogenized model. Therefore, under the present assumptions, omitting the seed layer as an explicitly separate phase does not materially alter the effective constitutive response.

Based on these considerations, the barrier and seed layers were not modeled as independent phases in the final RVE homogenization framework.

1. **Effect of the SiO_2_ Insulation Layer on RVE Effective Properties**

Unlike the barrier and seed layers, the SiO_2_ insulation liner has a substantially larger thickness and a strong constitutive contrast relative to both Cu and Si. Its influence on the homogenized effective properties must therefore be explicitly quantified.

To this end, matched RVE cases were analyzed with and without a 700 nm SiO_2_ liner[S6], and the resulting effective properties were compared. FIG. N5.1 summarizes the relative errors of all effective-property components using box plots, thereby showing not only the average deviation but also the spread and maximum deviation across different RVEs.


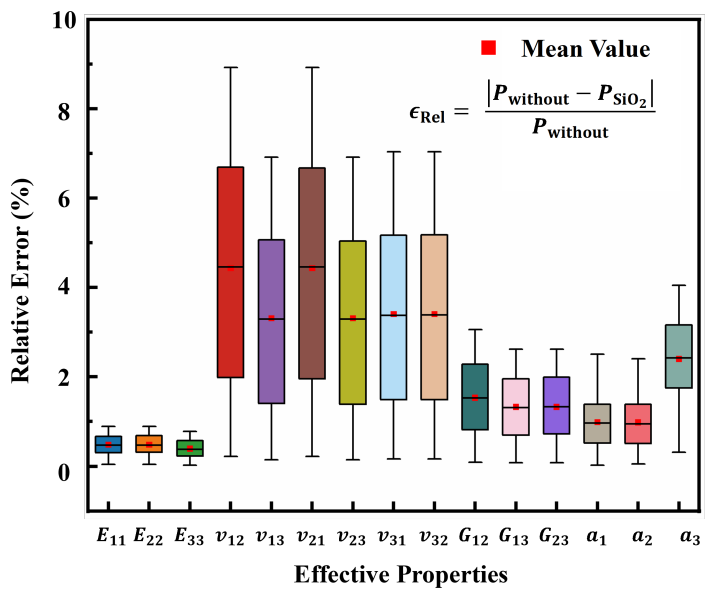


**FIG. N5.1.** Box plots of the relative errors in all 15 effective properties when the SiO_2_ insulation layer is omitted from the RVE homogenization. The relative error is defined as $\frac{\left| \text{P}_{\text{without}}\text{−}\text{P}_{\text{with}} \right|}{\text{P}_{\text{with}}}\text{×}\text{100\%}$. The red square marker indicates the mean value.

For the normal elastic modulus (*E*_11_, *E*_22_, and *E*_33_), neglecting the SiO_2_ layer results in only small deviations. The mean relative error is about 0.48% for the in-plane Young’s modulus and 0.39% for the out-of-plane modulus *E*_33_, while the maximum relative errors remain below 0.9%. By contrast, the omission of SiO_2_ has a more pronounced effect on the multi-axial coupling terms. For the Poisson’s ratios (*v*_12_, *v*_13_, *v*_21_, *v*_23_, *v*_31_, and *v*_32_), the mean relative errors increase to approximately 3.3%-4.4%, with maximum deviations reaching 6.9%-8.9%. The shear modulus (*G*_12_, *G*_13_, and G_23_) also show non-negligible deviations, with maximum relative errors up to 3.06%.

The effect on thermal expansion is particularly important. The in-plane coefficients of thermal expansion ($\alpha_{\text{1}}$ and $\alpha_{\text{2}}$) exhibit mean relative errors of around 0.98% when SiO_2_ is neglected, whereas the out-of-plane coefficient $\alpha_{\text{3}}$ shows a much larger mean relative error of 2.4%, with a maximum deviation of 4.0%. These results indicate that the SiO_2_ layer cannot be regarded as negligible in the present homogenization problem, especially when the objective is to predict wafer-level thermo-mechanical deformation.

1. **First-order Propagation of Effective Property Errors to Wafer Warpage.**

To interpret how RVE-level constitutive errors may affect macroscopic warpage, a first-order sensitivity analysis was carried out using a modified Stoney-type framework. This analysis is intended only as a qualitative pre-bifurcation sensitivity ranking under small-deflection conditions. It is not used as a quantitative predictor of post-bifurcation deformation.

Consider a simplified bilayer system consisting of a homogenized TSV interposer layer of thickness $\text{h}_{\text{f}}$ bonded to a silicon substrate of thickness $\text{h}_{\text{s}}$. Under a uniform temperature change $\text{∆}\text{T}$, the thermal mismatch between the two layers induces wafer curvature. Assuming $\text{h}_{\text{f}}\text{≪}\text{h}_{\text{s}}$ and small deflection, the curvature $\text{κ}$ is given by:

$\text{κ}\text{=}\frac{\text{6}\text{σ}_{\text{f}}\text{h}_{\text{f}}}{\hat{\text{E}_{\text{s}}}\text{h}_{\text{s}}^{\text{2}}}$ (*S8*)

where $\text{σ}_{\text{f}}$ is the biaxial film stress and $\hat{\text{E}_{\text{s}}}\text{=}\text{E}_{\text{s}}\text{/(1−}\text{v}_{\text{s}}\text{)}$ is the biaxial modulus of the silicon substrate.

Under an in-plane isotropic approximation, the thermally induced film stress can be written as:

$\text{σ}_{\text{f}}\text{=}\frac{\text{E}_{\text{eff}}}{\text{1−}\text{v}_{\text{eff}}}\text{(}{\text{α}_{\text{eff}}\text{−}\text{α}}_{\text{s}}\text{)∆}\text{T}$ (*S9*)

where $\text{E}_{\text{eff}}$, $\text{v}_{\text{eff}}$, and $\text{α}_{\text{eff}}$ are the homogenized effective Young’s modulus, Poisson’s ratio, and CTE of the TSV interposer layer, respectively, and $\text{α}_{\text{s}}$ is the CTE of the silicon substrate. The edge warpage *w* at wafer radius *R* is related to curvature by:

$\text{w}\text{=}\frac{\text{κ}\text{R}^{\text{2}}}{\text{2}}$ (*S10*)

Combining the above relations gives:

$\text{w}\text{=}\frac{\text{3}\text{R}^{\text{2}}\text{h}_{\text{f}}\text{∆}\text{T}}{\hat{\text{E}_{\text{s}}}\text{h}_{\text{s}}^{\text{2}}}\text{×}\frac{\text{E}_{\text{eff}}}{\text{1−}\text{v}_{\text{eff}}}\text{(}{\text{α}_{\text{eff}}\text{−}\text{α}}_{\text{s}}\text{)}$ (*S11*)

where

$\text{C=}\frac{\text{3}\text{R}^{\text{2}}\text{h}_{\text{f}}\text{∆}\text{T}}{\hat{\text{E}_{\text{s}}}\text{h}_{\text{s}}^{\text{2}}}$ (*S12*)

$\text{C}$ is a geometric and loading-dependent constant.

Applying logarithmic differentiation yields the first-order sensitivity coefficients:

$\text{S}_{\text{E}}\text{=}\frac{\text{∂ln}\text{w}}{\text{∂ln}\text{E}_{\text{eff}}}\text{=1}$ (*S13*)

$\text{S}_{\text{α}}\text{=}\frac{\text{∂ln}\text{w}}{\text{∂ln}\text{α}_{\text{eff}}}\text{=}\frac{\text{α}_{\text{eff}}}{{\text{α}_{\text{eff}}\text{−}\text{α}}_{\text{s}}}$ (*S14*)

$\text{S}_{\text{v}}\text{=}\frac{\text{∂ln}\text{w}}{\text{∂ln}\text{v}_{\text{eff}}}\text{=}\frac{\text{v}_{\text{eff}}}{{\text{1−}\text{v}}_{\text{eff}}}$ (*S15*)

Accordingly, the first-order relative warpage error may be approximated as:

$\frac{\text{∆}\text{w}}{\text{w}}\text{≈}\text{S}_{\text{E}}\text{(}\frac{\text{∆}\text{E}}{\text{E}_{\text{eff}}}\text{)+}\text{S}_{\text{α}}\text{(}\frac{\text{∆}\text{α}}{\text{α}_{\text{eff}}}\text{)+}\text{S}_{\text{v}}\text{(}\frac{\text{∆}\text{v}}{\text{v}_{\text{eff}}}\text{)}$ (*S16*)

In this simplified pre-bifurcation framework, the shear modulus does not appear as a first-order driving term. The key implication is that CTE errors are expected to be the most strongly amplified, because the thermal driving force depends on the mismatch (${\text{α}_{\text{eff}}\text{−}\text{α}}_{\text{s}}$), which is typically much smaller than $\text{α}_{\text{eff}}$ itself. By contrast, errors in $\text{E}_{\text{eff}}$ are transferred linearly, and the contribution of $\text{v}_{\text{eff}}$ is attenuated by the factor $\frac{\text{v}_{\text{eff}}}{{\text{1−}\text{v}}_{\text{eff}}}$, which is usually smaller than unity. This ranking is consistent with the numerical observation that the omission of SiO_2_ affects thermal-expansion-related quantities more strongly than the normal elastic modulus.

1. **Effect of the SiO_2_ Insulation Layer on Wafer-level Warpage Prediction.**

To quantify the wafer-level consequence of neglecting the SiO_2_ liner, warpage predictions were compared for matched wafer cases without (Wafer A) and with the SiO_2_ layer (Wafer B), as summarized in Table N5.1. In most cases, omitting the SiO_2_ layer leads to a systematic overestimation of wafer warpage, indicating that the simplified model exaggerates the effective thermo-mechanical mismatch of the TSV region.

**Table N5.1.** Comparison of wafer-level warpage for matched cases without (Wafer A) and with (Wafer B) the SiO_2_ insulation liner at different TSV counts per die.

|  | **2025 TSVs** | **7075 TSVs** | **11000 TSVs** | **12150 TSVs** | **20250 TSVs** | **30375 TSVs** |
| --- | --- | --- | --- | --- | --- | --- |
| Warpage of Wafer A (mm) | 0.39 | 0.82 | 0.98 | 2.20 | 4.19 | 6.56 |
| Warpage of Wafer B (mm) | 0.33 | 0.79 | 1.19 | 1.54 | 3.66 | 6.00 |

FIG. N5.2 and FIG. N5.3 illustrate the evolution of warpage modes as the total TSV count increases. An apparent inversion is observed for the 11000 TSVs case, where the model including SiO_2_ predicts a larger warpage magnitude than the model without SiO_2_. This isolated reversal should not be interpreted as evidence that the SiO_2_ layer is negligible. Rather, it suggests that omission of SiO_2_ may alter not only the constitutive magnitude of the homogenized TSV region, but also the predicted deformation-mode transition behavior.


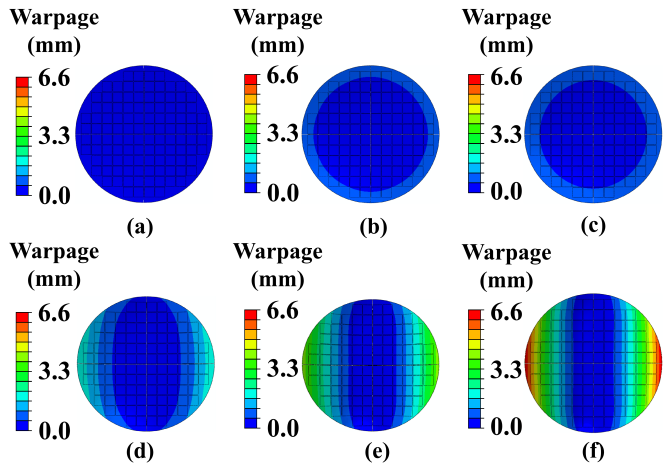


**FIG. N5.2.** Evolution of warpage deformation across the 12-inch wafer with increasing TSV density on interposer dies with uniform TSV distribution (without SiO_2_ layer). (a) 2025 TSVs, (b) 7075 TSVs, (c) 11000 TSVs, (d) 12150 TSVs, (e) 20250 TSVs, (f) 30375 TSVs.


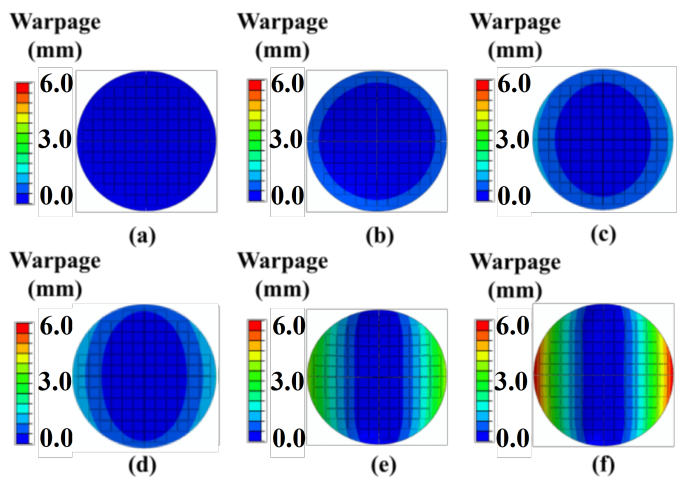


**FIG. N5.3.** Evolution of warpage deformation across the 12-inch wafer with increasing TSV density on interposer dies with uniform TSV distribution (with SiO_2_ layer). (a) 2025 TSVs, (b) 7075 TSVs, (c) 11000 TSVs, (d) 12150 TSVs, (e) 20250 TSVs, (f) 30375 TSVs.

A plausible explanation is that the simplified model shifts the effective bifurcation threshold by perturbing the homogenized elastic coupling, especially the effective Poisson’s ratios[S7]. In this sense, the 11000 TSVs case is more appropriately interpreted as a bifurcation-sensitive regime in which a small constitutive perturbation can change the apparent transition point between deformation modes. At the current stage, this mechanism should be regarded as a physically plausible interpretation rather than a rigorously proven post-bifurcation theory, because a full verification would require a dedicated nonlinear bifurcation analysis beyond the scope of the present study.

Overall, the wafer-level comparisons confirm the same conclusion obtained from the RVE-level analysis: while the barrier and seed layers can be omitted under the present assumptions with negligible first-order consequence, the SiO_2_ insulation liner introduces non-negligible deviations in the homogenized effective properties, especially in the coupling terms and thermal expansion coefficients, and can therefore affect both the predicted warpage magnitude and the apparent mode-transition behavior. For this reason, the final RVE data set and surrogate-model development in this work explicitly retain the 700 nm SiO_2_ layer.

**Note S6. Sensitivity to RVE Discretization**

Because the die-level validation in the main text was performed using a fixed RVE partition, an additional sensitivity study was conducted to verify that the reported agreement is not an artifact of a particular discretization choice. To this end, three representative test layouts with distinct TSV spatial organizations were examined, namely a top-heavy layout, a staircase-like layout, and a top-right localized layout (FIG. N6.1).

For each layout, the die domain was re-discretized using three RVE partition sizes, 80 $\text{μ}$m $\text{×}$ 80 $\text{μ}$m, 120$\text{ }\text{μ}$m$\text{ }\text{×}$ 120 $\text{μ}$m, and 240 $\text{μ}\text{m }\text{×}\text{ }$240 $\text{μ}$m, while all other modeling conditions, including the material system, thermal loading, boundary conditions, and homogenization workflow, were kept unchanged. The deformation predictions obtained under different partition sizes were then compared at both the global and field levels. At the global level, the response was characterized by the maximum deformation. Compared to the 120 $\text{μ}$m $\text{×}$ 120 $\text{μ}$m partition, the relative errors in the maximum deformation values were all within 0.1%.


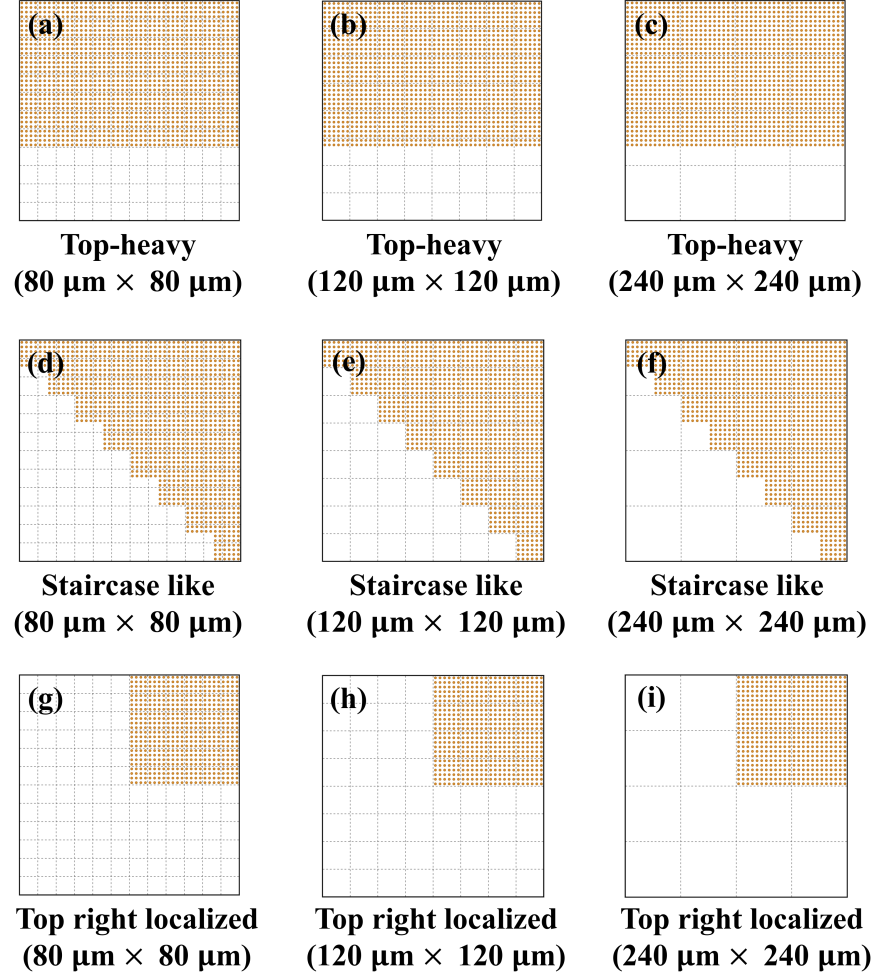


**FIG. N6.1.** Representative test layouts used in the die-level discretization sensitivity study under three RVE partition sizes: (a-c) top heavy layout discretized with 80 $\text{μ}$m $\text{×}$ 80 $\text{μ}$m, 120 $\text{μ}$m $\text{×}$ 120 $\text{μ}$m, and 240 $\text{μ}$m $\text{×}$ 240 $\text{μ}$m partitions, respectively, (d-f) staircase like layout under the same three partition sizes, and (g-i) top right localized layout under the same three partition sizes.

At the field level, the sensitivity to partition choice was quantified by comparing the displacement fields obtained under the 80 $\text{μ}$m $\text{×}$ 80 $\text{μ}$m and 240 $\text{μ}$m $\text{×}$ 240 $\text{μ}$m discretizations against the 120 $\text{μ}$m $\text{×}$ 120 $\text{μ}$m case, using RMSE and NRMSE, and further visualized by 2D top-view error elevation maps to show the spatial distribution of the inter-discretization discrepancies. The quantitative results are summarized in Table. N6.1 and FIG. N6.2. Overall, the fluctuation range remains very limited across all three layouts, indicating that the proposed framework is robust to reasonable changes in die-level RVE discretization and that the main validation conclusions are not sensitive to the specific partition choice adopted in the paper.

**Table N6.1.** Inter-discretization variation of the predicted deformation field for three representative test layouts under different die-level RVE partition sizes, quantified by RMSE and NRMSE relative to the 120 $\text{μ}$m $\text{×}$ 120 $\text{μ}$m.

|  | **RVE Size** | **RMSE (**$\text{μ}$**m)** | **NRMSE** |
| --- | --- | --- | --- |
| **Top-heavy** | (80 $\text{μ}$m $\text{×}$ 80 $\text{μ}$m) | 0.0048 | 0.23% |
|  | (240 $\text{μ}$m $\text{×}$ 240 $\text{μ}$m) | 0.0112 | 0.52% |
| **Staircase like** | (80 $\text{μ}$m $\text{×}$ 80 $\text{μ}$m) | 0.0030 | 0.15% |
|  | (240 $\text{μ}$m$\text{ ×}$ 240 $\text{μ}$m) | 0.0122 | 0.61% |
| **Top-right localized** | (80 $\text{μ}$m $\text{×}$ 80 $\text{μ}$m) | 0.0003 | 0.02% |
|  | (240 $\text{μ}$m$\text{ ×}$ 240 $\text{μ}$m) | 0.0009 | 0.05% |


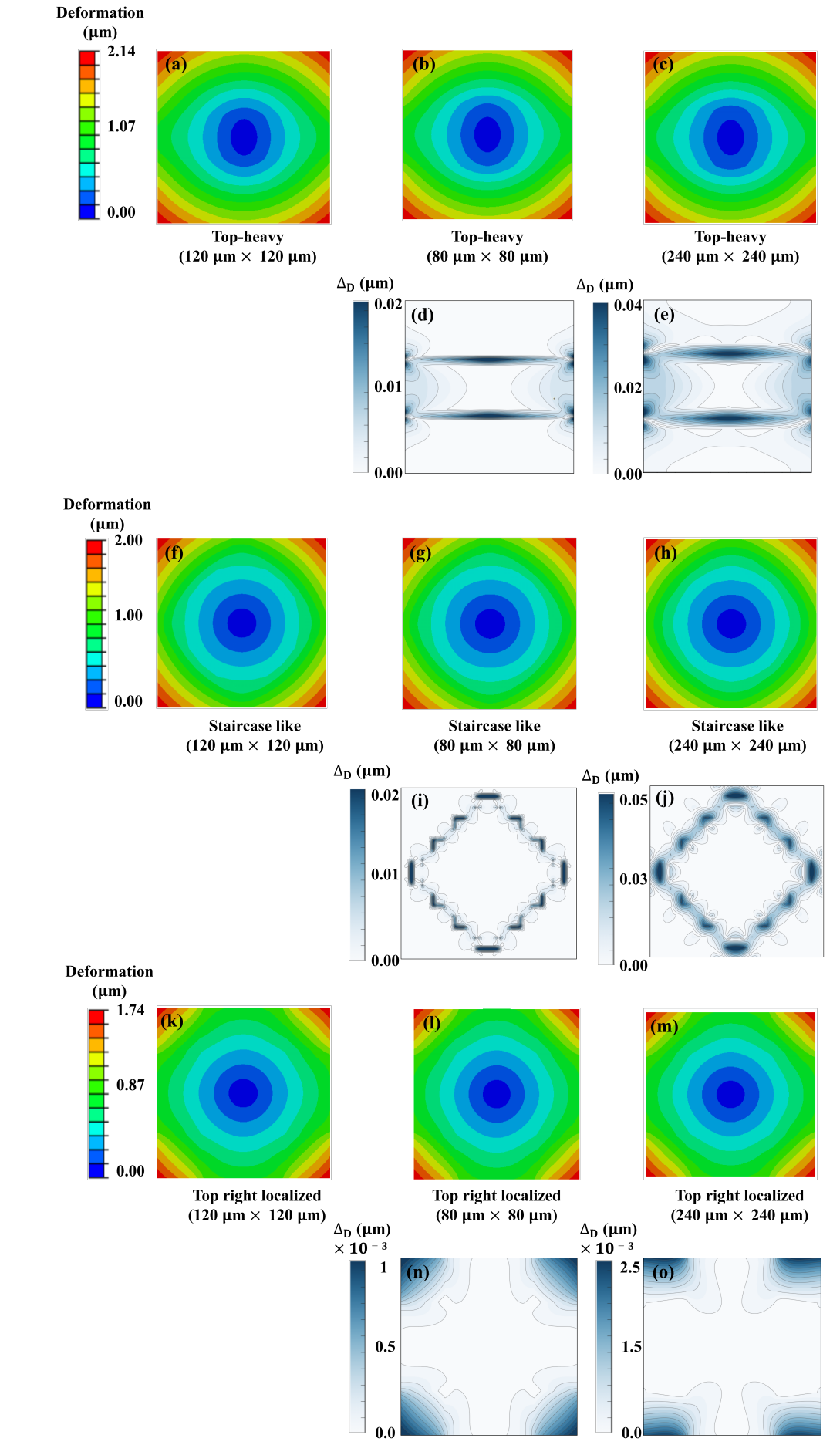


**FIG. N6.2.** Comparison of deformation predictions under different die-level RVE partition sizes for three representative test layouts. (a-c), (f-h), and (k-m) show the predicted deformation contours for the top heavy, staircase like and top right localized layouts, respectively, using 120 $\text{μ}$m$\text{ ×}$ 120 $\text{μ}$m, 80 $\text{μ}$m$\text{ ×}$ 80 $\text{μ}$m, and 240 $\text{μ}$m$\text{ ×}$ 240 $\text{μ}$m partitions. (d-e), (i-j), and (n-o) show the corresponding 2D top-view error elevation maps of the 80 $\text{μ}$m$\text{ ×}$ 80 $\text{μ}$m and 240 $\text{μ}$m$\text{ ×}$ 240 $\text{μ}$m cases relative to the 120 $\text{μ}$m$\text{ ×}$ 120 $\text{μ}$m reference, highlighting the spatial distribution of the inter-discretization discrepancies.

**TABLE S1.** **Detailed architectural configuration of the proposed TSV-INet.**

| **Network Component** | **Architecture Details** | **Key Hyperparameters & Dimensions** |
| --- | --- | --- |
| CNN Branch | ResNet-18 | Output feature dim = 15 |
| GNN Branch | 5-layer GINE | Hidden dim = 128  Dropout = 0.35  Readout = Global mean pool  Output feature dim = 15 |
| Global Descriptor | Global condition vector | Input dim = 9 |
| Fusion Head | 3-layer MLP concatenating features [**z**_c_, **z**_g_, ***g***] | Input dim = 39 (15+15+9)  Hidden layers = [256,128]  Final Output dim = 15 |

**TABLE S2. Detailed feature representations in the CNN and GNN branches of TSV-INet.**

| **Branch** | **Feature Level** | **Feature Contents** | **Data Dimension** | **Description / Physical Meaning** |
| --- | --- | --- | --- | --- |
| CNN | Pixel-level  (Grid map) | Young's Modulus | Matrix ($\text{256 × 256}$) | Spatial distribution of macroscopic equivalent material properties assigned to each pixel in the RVE |
|  |  | CTE | Matrix ($\text{256 × 256}$) |  |
|  |  | Poisson’s Ratio | Matrix ($\text{256 × 256}$) |  |
| GNN | Node-level  (Per TSV, 9D) | TSV center coordinates | Tensor ($\text{1 × 2}$) | (*x*, *y*) normalized coordinates |
|  |  | Radius metrics | Tensor ($\text{1 × 2}$) | *r* and *r*^2^ |
|  |  | Spatial location | Tensor ($\text{1 × 2}$) | Distance to RVE center and nearest boundary |
|  |  | Local neighborhood stats | Tensor ($\text{1 × 3}$) | Mean kNN distance, local packing density, and local anisotropy |
|  | Edge-level  (Per *k*NN pair, 7D) | Euclidean distance | Scalar | Normalized distance between two TSVs. |
|  |  | Relative direction | Tensor ($\text{1 × 2}$) | cos$\text{θ}$ and sin$\text{θ}$ of the connection vector |
|  |  | Radius variations | Scalar | Absolute difference $\left\vert\text{r}_{\text{i}}\text{−}\text{r}_{\text{j}} \right\vert$ |
|  |  | Midpoint geometry | Tensor ($\text{1 × 2}$) | Distance from edge midpoint to RVE center. |
|  |  | Connection rank | Scalar | Rank of the edge in the *k*-NN search. |
|  | Global-level  (Entire RVE, 9D) | Metal volume fraction | Scalar | Accurately integrated area of TSVs within the RVE. |
|  |  | Normalized TSV count | Scalar | *N*/60. |
|  |  | Global scale stats | Tensor ($\text{1 × 6}$) | Mean and Std of radius *r*, center distance *d*, and nearest-neighbor distance. |
|  |  | Global anisotropy | Scalar | Variance-based global geometric anisotropy. |

**TABLE S3. Comparison of representative related studies and the present work**

| **Criterion** | **ML-based surrogate studies in advanced packaging** | | | **Direct TSV-related studies** | |
| --- | --- | --- | --- | --- | --- |
|  | Jang *et al*.[S8] | Zhang *et al*.[S3], Wu *et al*.[S9], Mo *et al*.[S10] | Zhao *et al*.[S11] | Wu *et al*.[S12] | **This work** |
| **Year** | 2025 | 2023-2025 | 2025 | 2023 | 2026 |
| **Target Structure** | HBM | RDL | FOWLP wafer | TSV wafer | TSV wafer |
| **Method** | VAE + GMM clustering | FEM-generated data + CNN | FEM-generated data + CNN | FEM-generated data + CNN | FEM-generated data + Hybrid CNN + GNN |
| **Topology-aware** | Implicit | Implicit | Implicit | Implicit | Explicit |
| **Input representation** | Layout image | Pixel-level material field | Chip layout image | Array-based grid image | Multi-modal data |
| **Prediction target** | Effective properties | Effective properties | Wafer warpage | Effective properties | Effective properties |
| **Arbitrary layout support** | Limited | No | No | No | Enhanced |
| **Main limitation** | Indirect topology treatment;  still not real-time for full-chip deployment | High demand for labeled data; limited generalization to unseen layouts | Restricted to special designed chip; no explicit topology modeling | Restricted to array-based TSV layouts | Still requires high-fidelity FEM-generated labels for training |

**Note:** While the referenced studies address varying packaging structures and physical targets, they are compared here to benchmark the present work against the representative methodological advancements in ML-assisted surrogate modeling for advanced packaging.

**TABLE S4.** **Material properties for TSV interposer simulations.**

| Material Name | Silicon | Copper | SiO_2_ |
| --- | --- | --- | --- |
| CTE (1/℃) | 2.6E^-6^ | 1.64E^-5^ | 0.7E^-6^ |
| Young’s Modulus (MPa) | 1.31E^5^ | 9.18E^4^ | 7E^4^ |
| Poisson’s Ratio | 0.28 | 0.34 | 0.17 |

|  |
| --- |


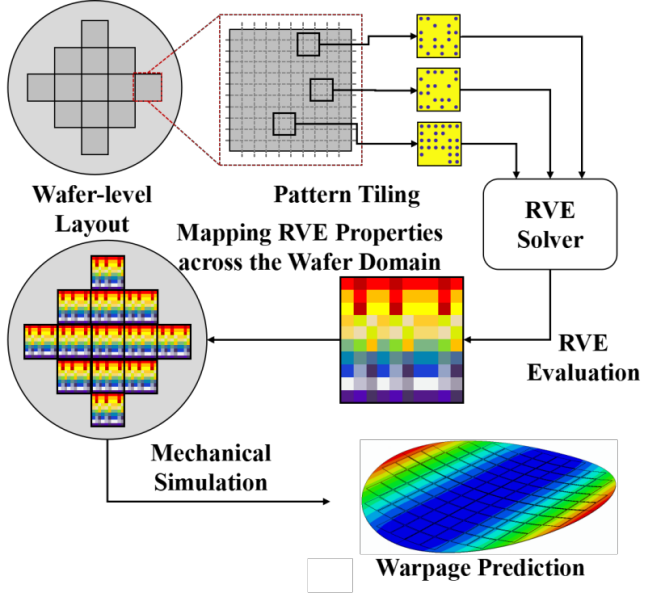


**FIG. S1.** Workflow for wafer warpage prediction using an RVE-based FEM approach.


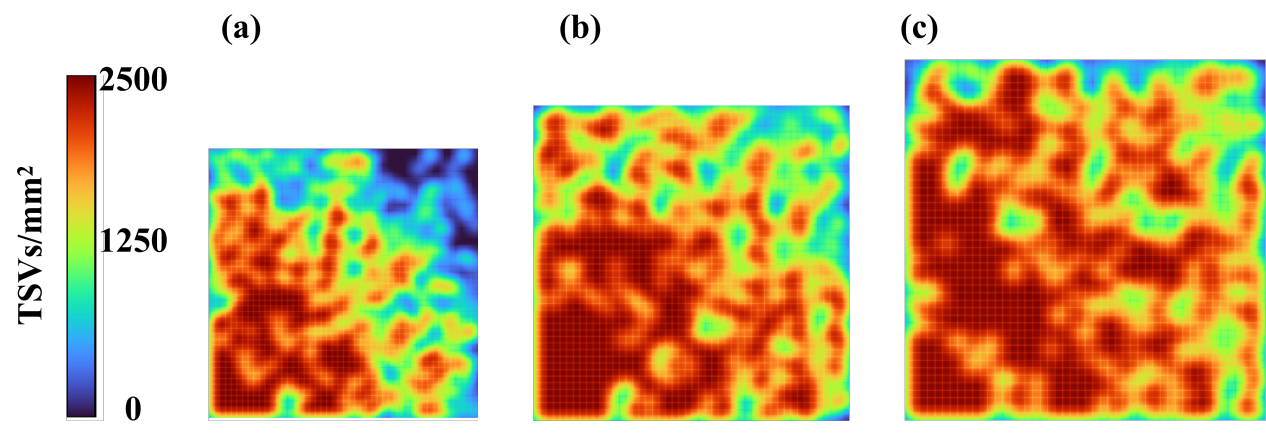


**FIG. S2**. The TSV density distribution of three different sized die patterns. (a) Case A, (b) Case B, (c) Case C.


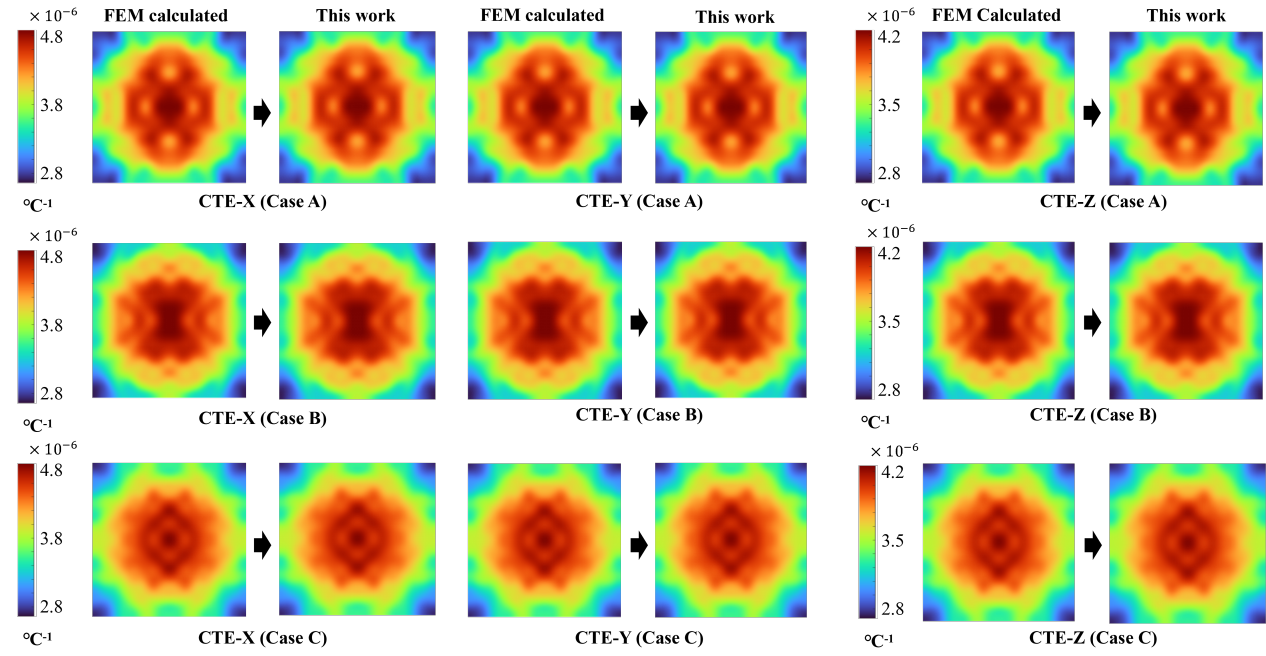


**FIG. S3**. The effective properties of RVE blocks in TSV patterns simulated by FEM simulation and TSV-INet prediction.


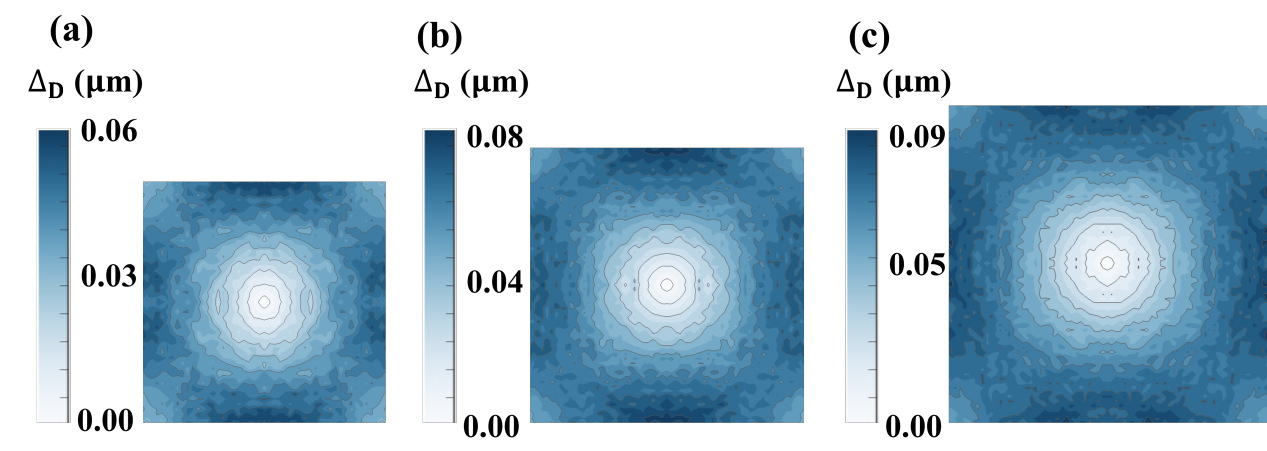


**FIG. S4**. 2D top-view error elevation maps of the die-level warpage prediction for the three test dies. (a) Case A (1.92 mm $\times$ 1.92 mm), (b) Case B (2.40 mm $\times$ 2.40 mm), (c) Case C (2.88 mm $\times$ 2.88 mm).


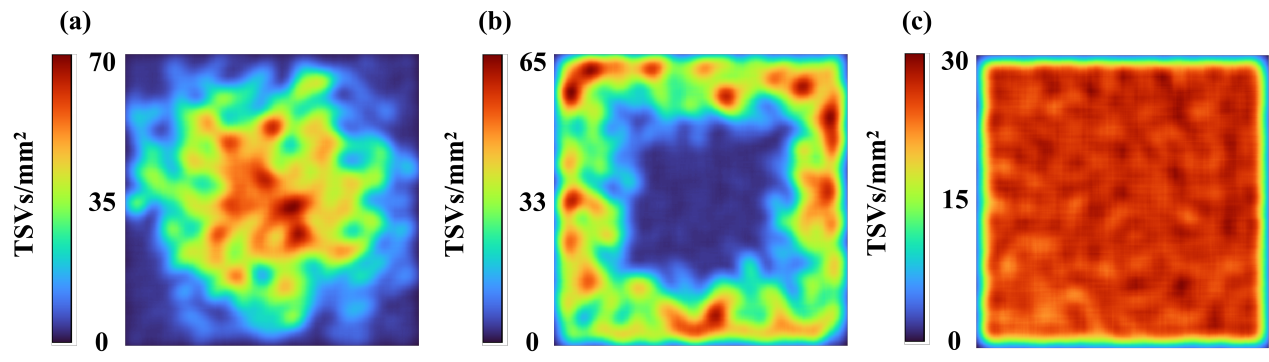


**FIG. S5**. The TSV density distribution of three different layout die patterns. (a) Center-concentrated pattern (Case X), (b) Perimeter-clustered pattern (Case Y), (c) Uniform pattern (Case Z).


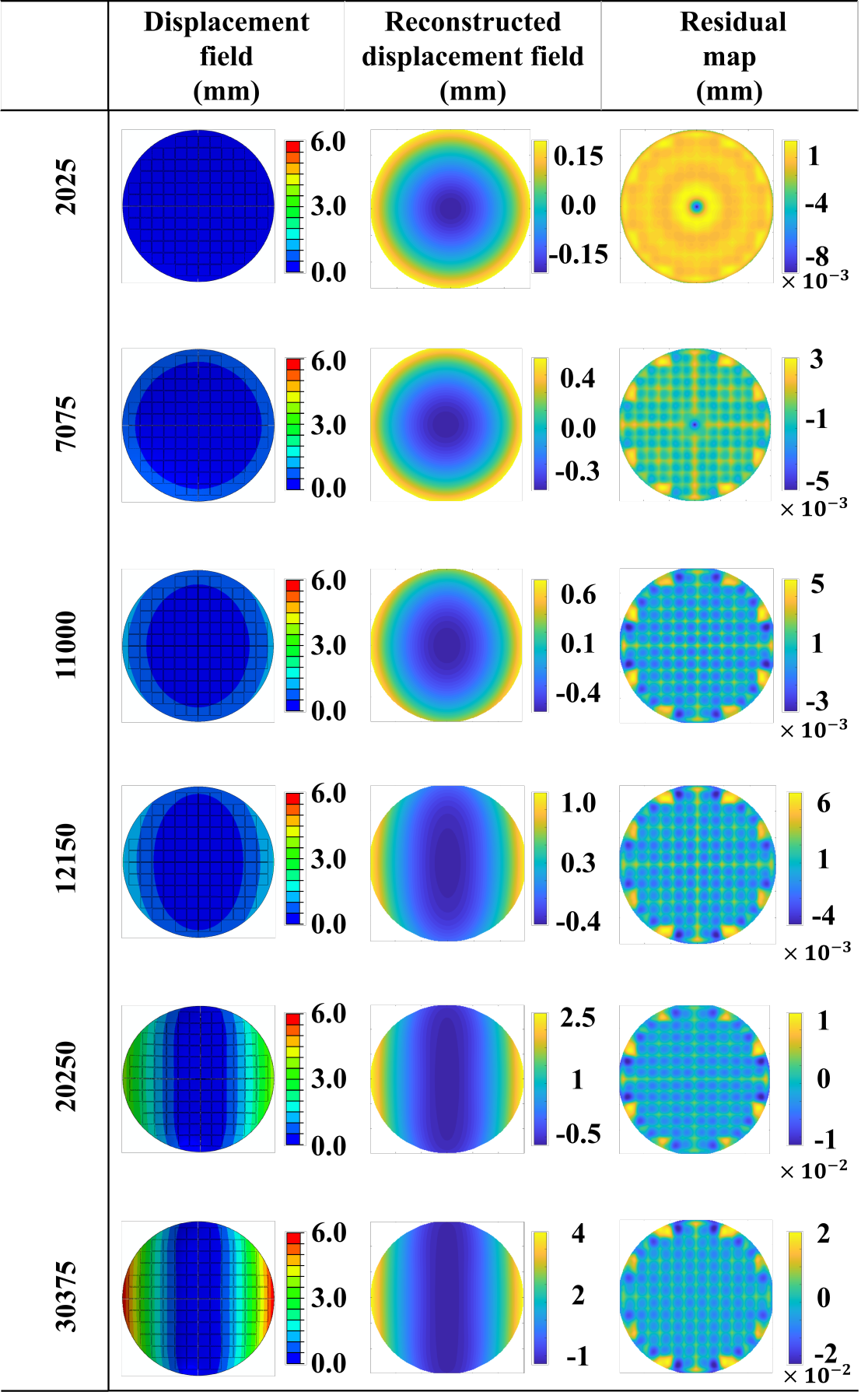


**FIG. S6**. Comparison of warpage contour maps and corresponding residual error maps between TSV wafers with varying TSV counts and Zernike-reconstructed wafer warpage.


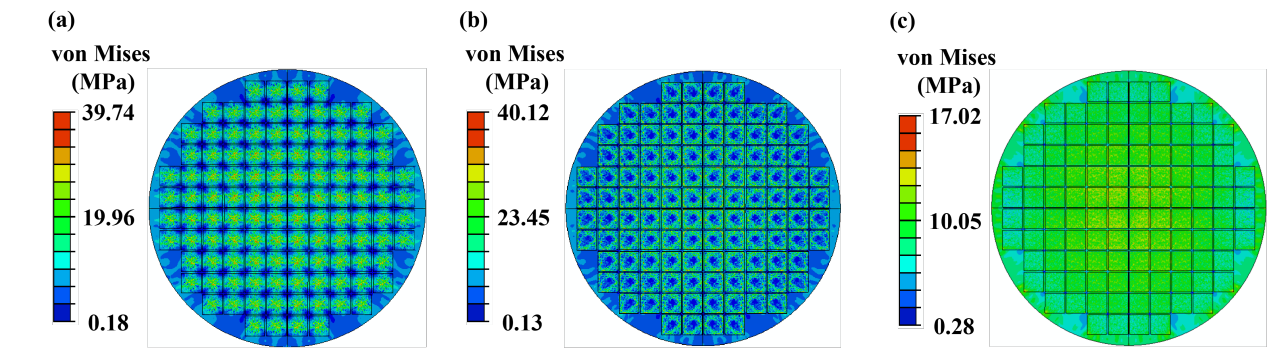


**FIG. S7**. Distribution of von Mises stress across the 12-inch wafer for the three TSV layout patterns. (a) Case X, (b) Case Y, (c) Case Z.


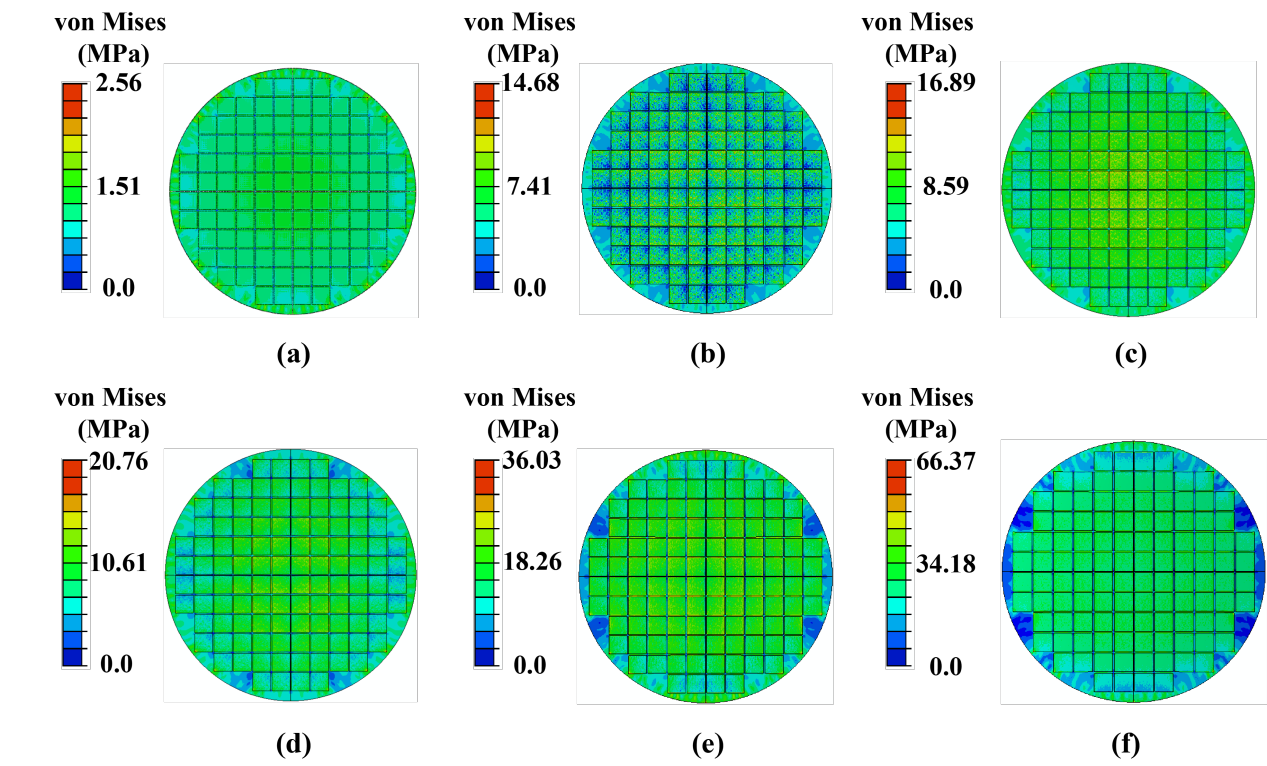


**FIG. S8.** Evolution of von Mises stress across the 12-inch wafer with increasing TSV density on interposer dies with uniform TSV distribution. (a) 2025 TSVs, (b) 7075 TSVs, (c) 11000 TSVs, (d) 12150 TSVs, (e) 20250 TSVs, (f) 30375 TSVs.


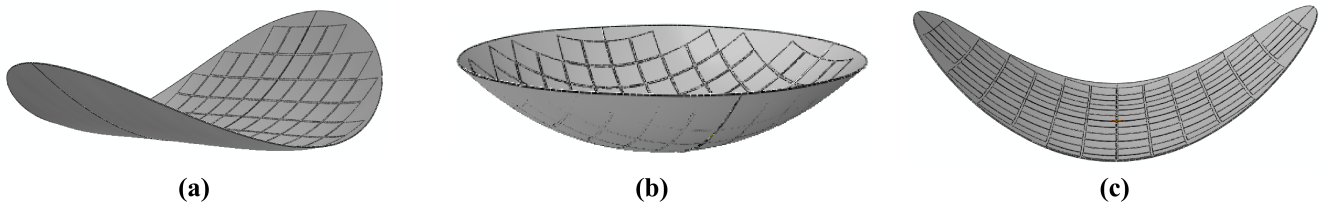


**FIG. S9.** The warpage modes associated with the three coefficients $\text{a}_{\text{2}\text{,−2}}$, $\text{a}_{\text{2}\text{,}\text{0}}$,$\text{a}_{\text{2}\text{,2}}$. (a) $\text{a}_{\text{2}\text{,−2}}$, (b) $\text{a}_{\text{2}\text{,}\text{0}}$, (c) $\text{a}_{\text{2}\text{,2}}$.


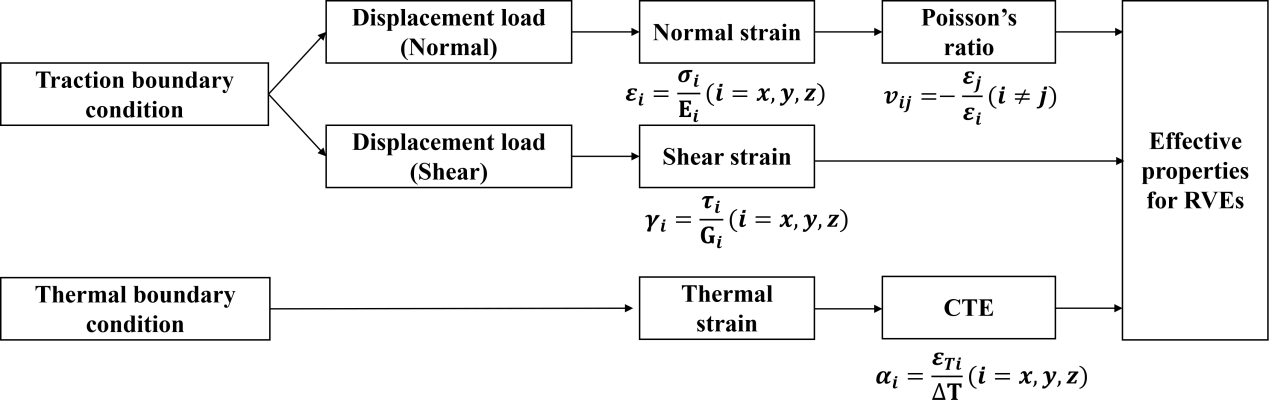


**FIG. S10.** The overall workflow of FEM-based material homogenization to get effective material properties for RVEs. Here, *i* and *j* denote the Cartesian directions (*i*, *j*$\text{∈}${*x*, *y*, *z*}). $\text{σ}_{\text{i}}$ and $\text{ε}_{\text{i}}$ are the normal stress and normal strain along direction *i*, and *E_i_* is the corresponding Young’s modulus. *v_ij_* is the Poisson’s ratio. $\text{τ}$ and $\text{γ}$ denote the shear stress and shear strain, respectively, and *G* is the shear modulus. For thermal loading, $\text{ε}_{\text{i}}^{\text{T}}$ represents the thermally induced strain in direction *i*, $\text{∆}\text{T}$ is the applied temperature change, and $\text{α}$ is the coefficient of thermal expansion of the RVE.


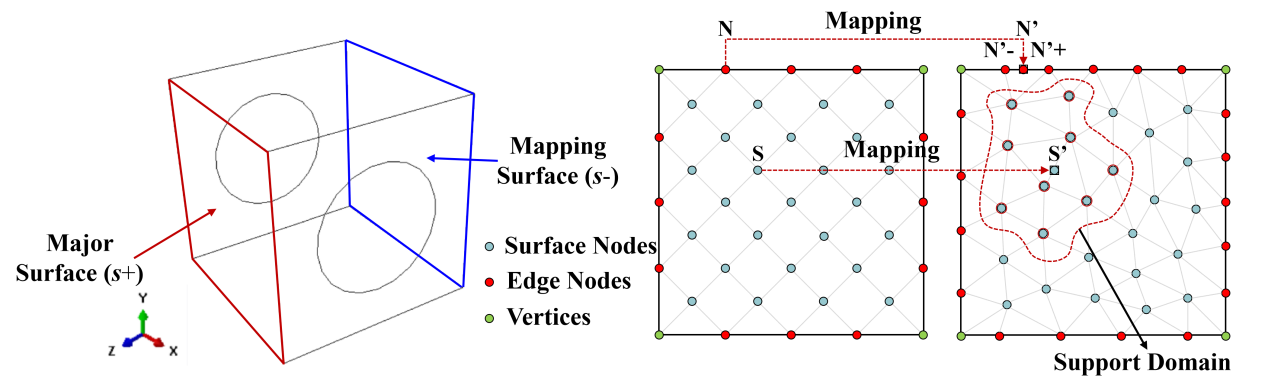


**FIG. S11.** Sketch of surface and edge interpolation. (a) Illustration of opposing faces under periodic boundary conditions, (b) Schematic of node correspondence based on RPIM interpolation.


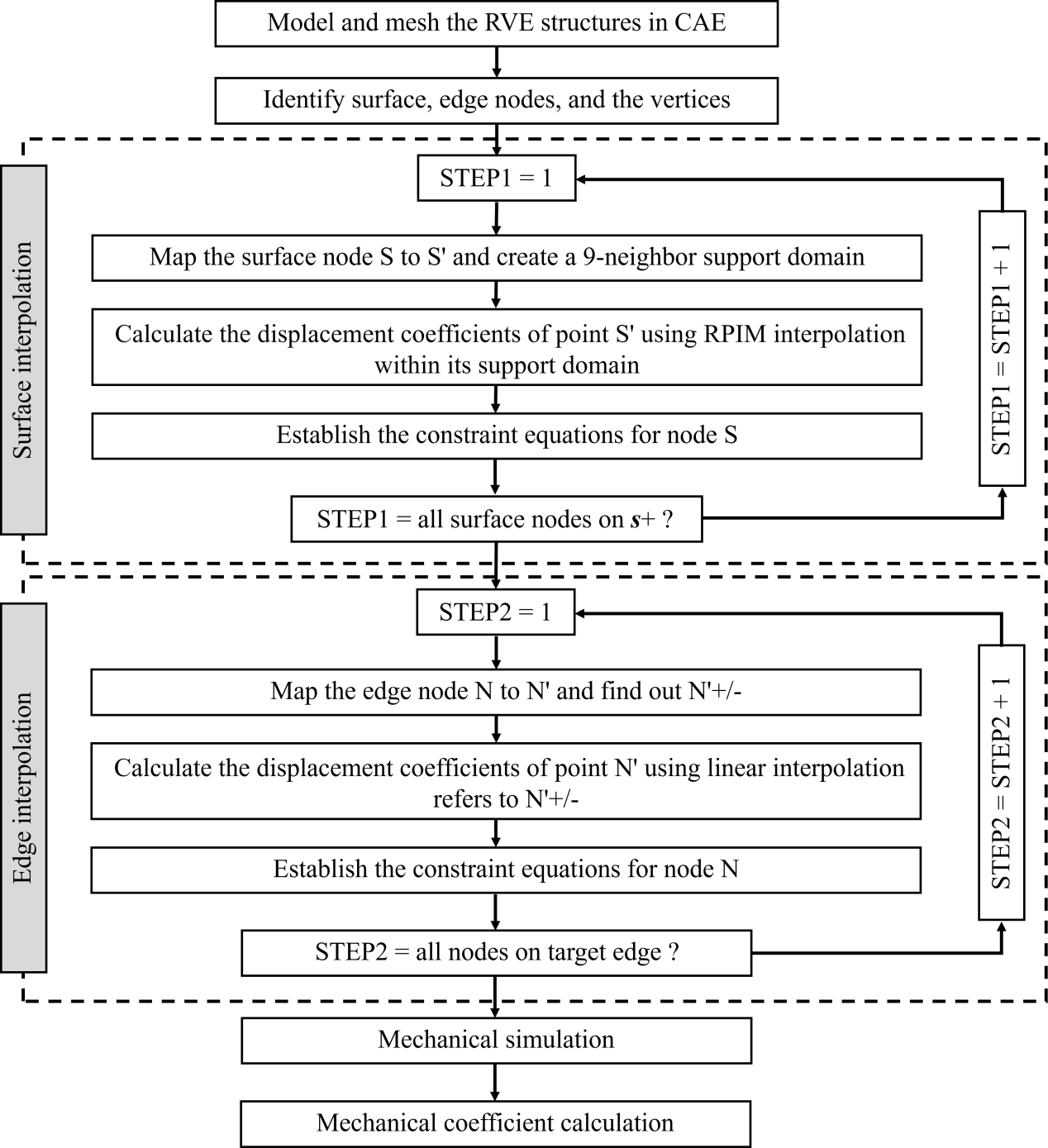


**FIG. S12.** Flowchart for applying periodic boundary conditions to asymmetric RVE structures.

**Reference**

[S1] Xu K., *et al*. How powerful are graph neural networks?. arXiv preprint arXiv. 1810.00826 (2018).

[S2] Hu W., *et al*. Strategies for pre-training graph neural networks. arXiv preprint arXiv. 1905.12265 (2019).

[S3] Zhang Y., et al. Unified Convolutional Neural Network Framework for Thermo-mechanical Modeling of Substrate with Multi-materials. 2025 26th International Conference on Electronic Packaging Technology (ICEPT). 1-6 (2025).

[S4] Li Y., *et al*. 3D microelectronic packaging: from fundamentals to applications. Springer, 57 (2017).

[S5] Liu T., *et al*. Study of the protrusion of through-silicon vias in dual annealing-CMP processes for 3D integration. Microsystems & Nanoengineering. 11(1): 25 (2025).

[S6] Xi Y., *et al*. The impact of sidewall copper grain condition on thermo-mechanical behaviors of TSVs during the annealing process. Microsystems & Nanoengineering. 10.1: 194 (2024).

[S7] Vinciguerra V. *et al*. Modelling the elastic energy of a bifurcated wafer: a benchmark of the analytical solution vs. the ANSYS finite element analysis. Composite Structures. 281: 114996 (2022).

[S8] Jang H., *et al*. Design-Aware Full-Chip Warpage Modeling for STCO: Bridging Reliability and Design for a New Era of Advanced Systems. 2025 Symposium on VLSI Technology and Circuits (VLSI Technology and Circuits), 1-3 (2025).

[S9] Wu X., *et al*. An RDL Modeling and Thermo-Mechanical Simulation Method of 2.5 D/3D Advanced Package Considering the Layout Impact Based on Machine Learning[J]. Micromachines 14(8), 1531 (2023).

[S10] Mo Q., *et al*. Cross-Scale Thermodynamic Simulation of RDL Using Hybrid Machine Learning and Finite Element Modeling. 2025 26th International Conference on Electronic Packaging Technology (ICEPT), 1-7 (2025).

[S11] Zhao, X., *et al*. Warpage prediction of fan-out wafer-level package based on coupled deep learning and finite element simulation. Microelectronics Reliability. 170: 115759 (2025).

[S12] Wu X. *et al*. TSV wafer warpage simulation and process induced strain prediction by machine learning-based anisotropic equivalent modeling method. 2023 IEEE 25th Electronics Packaging Technology Conference (EPTC). 780-786 (2023).
